# Auto-aggregation in *Streptococcus intermedius* is driven by the Pel polysaccharide

**DOI:** 10.1128/mbio.01196-25

**Published:** 2025-07-07

**Authors:** Deepa Raju, Siobhán A. Turner, Karla Castro, Gregory B. Whitfield, Daphnée Lamarche, Sahil Mahajan, Roland Pfoh, Stephanie H.W. Chuang, François Le Mauff, Maju Joe, Susmita Sarkar, Todd L. Lowary, Donald C. Sheppard, Daniel J. Wozniak, Michael G. Surette, P. Lynne Howell

**Affiliations:** 1 Program in Molecular Medicine, The Hospital for Sick Children 7979, Toronto, Ontario, Canada; 2 Department of Biochemistry, University of Toronto 7938, Toronto, Ontario, Canada; 3 Department of Biochemistry and Biomedical Sciences and the Michael G. DeGroote Institute for Infectious Disease Research, McMaster University 3710, Hamilton, Ontario, Canada; 4 Department of Microbial Infection and Immunity, The Ohio State University 2647, Columbus, Ohio, USA; 5 Department of Microbiology and Immunology and Medicine, McGill University 5620, Montreal, Québec, Canada; 6 Department of Medicine, McGill University 5620, Montreal, Canada; 7 McGill Interdisciplinary Initiative in Infection and Immunity, Montreal, Canada; 8 Department of Chemistry, University of Alberta 3158, Edmonton, Alberta, Canada; 9 Institute of Biological Chemistry, Academia Sinica, and Institute of Biochemical Sciences, National Taiwan University, Taipei, Taiwan; The University of Kansas Medical Center, Kansas City, Kansas, USA

**Keywords:** Biofilm, *S. intermedius*, Pel exopolysaccharide, aggregation, infection, host immune responses

## Abstract

The Streptococcus milleri group (SMG), comprising *Streptococcus intermedius, anginosus*, and *constellatus*, can asymptomatically colonize various mucosal sites of healthy individuals. These bacteria are opportunistic pathogens that cause different types of infections across various anatomical sites. Although the pathogenic mechanisms leading to infections are not well defined in the SMG, auto-aggregation is a key driver of biofilm adhesion and cohesion in many Streptococci and Staphylococci. Here, we identify a *S. intermedius* gene cluster with significant homology to the *Bacillus cereus* and *Pseudomonas aeruginosa pel* operons, which are required for Pel exopolysaccharide production and biofilm formation in these species. This cluster contains five genes that are homologous to *pelDEA_DA_FG* in other gram-positive species and four additional genes of unknown function. Characterization of a panel of clinical *S. intermedius* isolates identified a range of adherent biofilm and aggregation phenotypes , and aggregation in strain C1365 was dependent on each of the *pelDEA_DA_FG* genes. Deletion of two of the additional genes*, SIR_1592* and *SIR_1594*, reduced but did not abolish the aggregation phenotype. Furthermore, we demonstrate that SIR_1591 is a glycoside hydrolase and that C1365 produces a GalNAc-rich polymer as aggregates were disrupted by theα−1,4-*N-*acetylgalactosaminidases PelA and Sph3, but not the α−1,4-*N-*galactosaminidase Ega3. Using an abscess model of mouse infection, we show that loss of Pel production in a C1365 Δ*pelF* mutant allows for more effective bacterial clearance. The polymer also affects how *S. intermedius* interacts with the host immune system. Collectively, our data suggest that Pel biosynthesis contributes to *S. intermedius* pathogenicity.

**IMPORTANCE:**

SMG species are increasingly being recognized as pathogens. Despite their clinical relevance, little is known about how SMG members transition between asymptomatic colonization and infection. Herein, we show that clinical isolates of *S. intermedius* can be classified into four groups based on their aggregation and adherent biofilm phenotypes. We demonstrate that aggregation is dependent on the Pel polysaccharide and that Pel production allows bacteria not only to persist longer during infection but also modulates the immune responses of the host. Pel production requires the canonical *pelDEA_DA_FG* genes. We also identified four additional genes in the *S. intermedius pel* cluster and found that under the conditions tested, two of these genes play a role in aggregation and Pel production. Functional homologs of the additional genes play major roles in host-pathogen interactions and stress responses in other bacteria, suggesting that these additional genes could play a role in Pel-related infections.

## INTRODUCTION

Bacteria most commonly reside in multicellular communities called biofilms. Biofilms can form on medical devices, such as catheters, feeding tubes, and ventilators, as well as on mucosal surfaces within the body, and are predicted to be involved in ~80% of all chronic microbial infections (1–3). Biofilm-embedded bacteria are typically more tolerant to immunological, chemical, and mechanical perturbations than their planktonic counterparts (1, 4, 5). This makes the treatment of biofilm-associated infections extremely challenging, and the capacity to form a biofilm is a major virulence factor.

Biofilms can be surface-attached or exist as multicellular aggregates in suspension (6–8). There is increasing evidence to suggest that aggregates formed in media or host fluids exhibit the same characteristics as surface-attached biofilms and are increasingly being recognized for their role in pathogenicity (6, 9–12). Multiple infections have been linked to aggregates, including those associated with cystic fibrosis (7, 13), dermal wounds (9, 11), and otitis media (14). Bacteria in both surface-attached biofilms and aggregates are recalcitrant to antibiotic treatment and are protected from the immune system. These advantages are enabled, at least in part, by the presence of an extracellular matrix made of various components including water, lipids, proteins, extracellular DNA (eDNA), RNA, membrane vesicles, phages, and exopolysaccharides.

One important matrix component of both gram-negative and gram-positive bacterial biofilms is the Pel polysaccharide (15–17). This polysaccharide has been well-studied in *Pseudomonas aeruginosa* (18) where it is required for pellicle formation at the air-liquid interface and the characteristic wrinkly colony morphology of *P. aeruginosa* (19, 20). Pel is also critical for cell-to-cell adhesion and the formation of aggregates (21). Within the extracellular matrix, Pel exists in two forms: a cell-associated and cell-free form. A recent study suggests that the production of the cell-free form contributes to the biomechanical properties of the biofilm and decreases the virulence of *P. aeruginosa* in *Drosophila melanogaster* and *Caenorhabditis elegans* infection models (19). Although the structure of the cell-associated form has yet to be determined, the cell-free form is a homopolymer of partially de-*N-*acetylated α−1,4-linked *N*-acetylgalactosamine (GalNAc), which consists predominantly of a dimeric repeat of GalNAc and galactosamine (GalN) (22). Deacetylation of the polymer renders Pel cationic, and this modification is required for biofilm formation (21). The cationic charge of Pel facilitates its interaction with eDNA (21) and host-derived anionic polymers in cystic fibrosis sputum (23); interactions that help stabilize the structural core of the biofilm (18, 23). Pel also interacts with protein components, such as CdrA, and enhances biofilm aggregation (24, 25). The presence of Pel in the extracellular matrix increases bacterial tolerance to antibiotic treatment; an effect that is further enhanced by its interaction with eDNA (23, 26).

**Fig 1:**
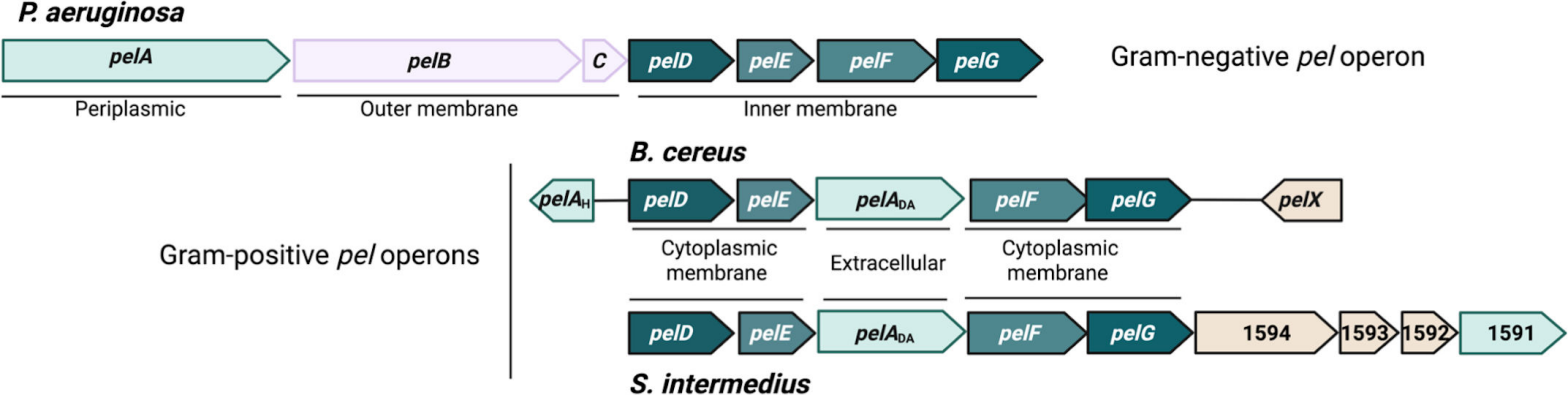
Model of the Pel biosynthetic complex in *S. intermedius.* Schematic representation of the *P. aeruginosa*, *B. cereus,* and *S. intermedius* (top) *pel* operons. Additional genes in the *pel* operons are indicated in wheat. Enzymes that are known or predicted to modify the Pel polymer, either via deacetylation or hydrolysis are shown in pale green. Proteins exclusive to the outer membrane of the Gram-negative *pel* operon, *pelB* and *pelC,* are in pink. In the *S. intermedius, pel* operon SIR_1591 to SIR_1594 are represented by their numbers 1591–1594. In the *P. aeruginosa* operon, *pelC* is represented by the letter C. The cellular location of the genes in the canonical *pel* operon is as indicated. The figure was created in BioRender.

All the genes in the *P. aeruginosa pelABCDEFG* operon are essential for biofilm and aggregate formation (Fig. 1) (27–31). PelDEFG forms an inner membrane complex that enables polymerization and export of the Pel polymer across the cytoplasmic membrane (32). This biosynthetic complex is activated by the binding of cyclic di-guanosine monophosphate (c-di-GMP) to PelD (33, 34), whereupon the glycosyltransferase PelF uses UDP-*N*-acetylgalactosamine as a substrate for Pel synthesis (28). PelE is a predicted protein interaction module, whereas PelG is the predicted translocase. PelB and PelC help guide the Pel polymer across the periplasm and are required for its export into the extracellular space (30, 35). PelA is a periplasmic modification enzyme that exhibits both glycoside hydrolase and deacetylase activities (19, 29, 36). These activities are required for the production of cell-free Pel and Pel deacetylation, respectively (19, 22, 29).

Using a computational pipeline that allows for the unbiased identification of functionally related gene clusters, we recently discovered a *pel-*like gene cluster, *pelDEA_DA_FG,* in a wide variety of gram-positive bacterial species, including Streptococci, and have demonstrated that the GalNAc-rich Pel-like polymer produced by this gene cluster is required for biofilm formation in *B. cereus* ATCC 10987 (15, 16). The *pel*-like gene cluster found in gram-positive bacteria contains the core *Pseudomonas* homologs, *pelDEFG,* and a truncated extracellular *pelA* homolog, referred to herein as *pelA_DA_*, which lacks the hydrolase domain found in the *P. aeruginosa* counterpart (Fig. 1). Although not a core part of the *pel* gene cluster, a glycoside hydrolase is frequently found divergently encoded upstream or downstream of the *pel* locus in gram-positive species (Fig. 1). In *B. cereus*, similar to *P. aeruginosa,* deletion of the upstream glycoside hydrolase resulted in increased biofilm biomass (16).

In Streptococcal genomes, including *S. intermedius*, the *pel*-like gene cluster diverges from the canonical gram-negative *P. aeruginosa pelABCDEFG* and gram-positive *B. cereus pelDEA_DA_FG* operons in a number of ways, including the presence of four additional genes (SIR 1591–1594 in *S. intermedius*) whose function in Pel biosynthesis and biofilm formation is currently unknown (Fig. 1). The most prominent difference in the core *pel* genes is in *pelD (15, 17). P. aeruginosa* and *B. cereus pelD* encode proteins, *Pa*PelD and *Bc*PelD, with four transmembrane (TM) helices, and cytoplasmic GAF and GGDEF domains, the latter of which binds c-di-GMP to regulate Pel biosynthesis (33, 34). In contrast, in *Streptococcus* spp., the *pelD* homolog encodes a highly divergent protein (17). *S. intermedius* PelD (*Si*PelD) has the TM and GAF domains but lacks the GGDEF domain. *Si*PelD also contains an additional N-terminal domain that is predicted to be a degenerate short-chain dehydrogenase/reductase (SDR) (17). The function of the degenerate SDR domain is currently unknown. The other canonical *pel* genes in the operon, *pelDEFG*, are predicted to perform similar functions in both the gram-negative and gram-positive species. PelF and PelG are the polymerase and predicted translocase, respectively, whereas PelE is a predicted protein interaction module. As found in *B. cereus, Si*PelA only consists of a deacetylase domain, and we have similarly designated the protein as *Si*PelA_DA_ (Fig. 1).

This divergent Pel gene cluster is found in the heterogeneous Viridans group Streptococci (16). The Streptococcus milleri/anginosus group (SMG), included in the Viridans group, consists of *Streptococcus intermedius*, *Streptococcus constellatus*, and *Streptococcus anginosus*. These species are known to colonize various mucosal sites such as the oral, gastrointestinal, and urogenital tracts of healthy individuals (37). However, with the advent of better detection and diagnostic techniques, their clinical relevance as pathogens is increasingly being recognized (38, 39). These bacteria have been isolated from various infection sites, including abscesses, pneumonia, pleural empyema, and the bloodstream (40–44), and are the most common cause of invasive pyogenic streptococcal infections in the Calgary Health Region between 1999 and 2004 (45, 46). SMG organisms are also of considerable concern for children as complications from sinusitis and otitis media, two common childhood infections, have resulted in a significant increase in intracranial abscesses requiring surgery in the last decade (47). These species have also been identified as significant pathogens during pulmonary exacerbation in individuals with cystic fibrosis (44, 48–53). Despite their clinical relevance, little is known about SMG pathogenicity and which environmental signals induce lifestyle transitions between asymptomatic colonization and infection.

Given the role of Pel in other species and the lack of any other identifiable exopolysaccharide biosynthetic operons, we hypothesized that Pel may play a role in biofilm formation in SMG species and thus contribute to their pathogenicity and virulence. Using *S. intermedius* as our model organism, we have established a role for Pel in aggregate formation in these species. Screening a panel of clinical *S. intermedius* isolates, we discovered a range of adherent biofilm and aggregation phenotypes across the 11 strains tested. We found that all the canonical *pel* genes are essential for aggregation and that the ability to produce the polymer is associated with the persistence of infection in a mouse abscess model and also modulates the immune responses of the host. Under the conditions tested, deletion of *SIR_1594* and *SIR_1592* resulted in reduced aggregation and altered levels of Pel production, suggesting that these genes play a role in these processes. We also demonstrate that *SIR_1591* is an active glycoside hydrolase and that the hyper-aggregating strain C1365 produces a predominantly GalNAc-rich polymer. Our data provide strong evidence that Pel is an important virulence factor in *S. intermedius*.

## MATERIALS AND METHODS

### Strains and media

*S. intermedius* strains were cultured at 37°C, 5% CO_2_ using THY broth (37 g/L Todd Hewitt Broth, 0.5% [wt/vol] yeast extract), or THY agar (37 g/L Todd Hewitt Broth, 0.5% [wt/vol] yeast extract, 1.5% agar). Antibiotic selection was performed by adding 250 µg/mL kanamycin as required. A list of the clinical strains used in this study and the source they were isolated from is presented in Table 1.

**TABLE 1:** List of *S. intermedius* strains used in this study

Strain designation	Anatomical source	Anatomical source	Production of pel	Reference
C1365	Blood	Calgary, AB, Canada	Yes	(48, 54)
C1369S	Blood	Calgary, AB, Canada	No	(48, 54)
C1369R	Blood	Calgary, AB, Canada	No	(48, 54)
C1377	Blood	Calgary, AB, Canada	Yes	(48, 54)
C1390	Brain	Calgary, AB, Canada	Yes	(48, 54)
C260	Invasive	Calgary, AB, Canada	Yes	(48, 54)
M60R	Lung Exacerbation	Calgary, AB, Canada	Yes	(48, 54)
B196	Hip abscess	Calgary, AB, Canada	No	(48, 54)
SAG05	Neck lymph node	Toronto, ON, Canada	Yes	This work
AP1	Pleural empyema	Mesa, Arizona, USA	Yes	(48, 54)
AP2	Pleural empyema	Mesa, Arizona, USA	No	(48, 54)

### Genome assembly

The SMG isolates isolated from healthy and acutely/chronically ill individuals were sequenced on the Illumina MiSeq platform by the McMaster Sequencing facility (Ontario, Canada) using Nextera DNA library preparation and standard protocols to generate 2 × 250 bp paired-end reads. Read quality was assessed via FastQC (Andrews Simon, 2010). Paired-end Illumina reads were *de novo* assembled using the a5 pipeline (55). Contigs smaller than 1,000 bp with an average coverage below 10-fold were excluded from the analysis. Contigs were ordered with Mauve Multiple Genome Alignment (build date Feb 13, 2015) (56) to the species reference, *S. intermedius* B196. Bacterial gene prediction and annotation were performed using Prokka v1.12-beta (57) using high-quality species-specific reference genomes (58). Bacteriophages gene prediction and annotation were performed using the RAST tool kit (59) via the PATRIC (Pathosystems Resource Integration Center) web-based interface (60).

### Generation of mutant strains

Mutants were generated by replacing the target gene with a cassette conferring resistance to kanamycin as described previously (61–63). Primers and plasmids used in the study are listed in Table S1. All primers were ordered from Sigma Aldrich. Briefly, the gene deletion allele was constructed by amplifying flanking regions of the ORF and a kanamycin cassette and fused together by overlap extension PCR using Phusion DNA polymerase. The DNA fragment containing the cassette and flanking sequences was then linearized by restriction digest, gel purified, and 50–100 ng of the purified fragment was added to overnight cultures diluted to OD_600_ ~0.08 in 3 mL THY and incubated for 1 h at 37°C, 5% CO_2_ before adding DNA and 500 ng/mL competence stimulating peptide (62, 63). Cultures were incubated for a further 3–4 h before plating on THY agar containing the relevant antibiotic. The deletion and presence of the antibiotic cassette were further confirmed by PCR. The knockout constructs encoding 1,000 bp upstream and downstream with a Kanamycin insertion for *pelF* and *SIR_1591* were designed, were codon-optimized, and were synthesized by BioBasic. All constructs were sequence verified by The Centre for Applied Genomics (TCAG) at The Hospital for Sick Children. Whole genome sequencing was performed using the long-read Oxford Nanopore sequencing technology (Plasmidsaurus). Sequence alignments, single nucleotide polymorphisms, and insertions were analyzed in Geneious by mapping the mutant genome sequences to the wild-type reference genome.

### Analysis of surface adherence

Surface adherence of the 11 clinical strains and the mutants was assessed using a crystal violet assay as described previously (64, 65). Overnight cultures were diluted to OD_600_ ~0.005 and incubated for 24 h. Unattached cells were removed by submerging the poly-lysine-coated polystyrene multi-well plates twice in water and discarding the run-off. Adherent cells were stained in 0.1% crystal violet for 30 min and excess stain was removed by submerging plates twice in water. Staining was quantified by eluting crystal violet in 30% acetic acid and measuring absorbance at 550 nm.

### Aggregation assays

Aggregation assays were performed after the *S. intermedius* strains were grown overnight at 37°C. Cultures were centrifuged at 3,000 × *g* for 5 min. Media was removed, and the cells were resuspended in 1 mL aggregation buffer (1 mM Tris, 2 mM CaCl_2_, 3 mM MgCl_2_, 150 mM NaCl, and buffered to pH 7.4 with HCl) or in 10% artificial sputum medium (ASM) (Bio Chemazone, AB, Canada) and centrifuged at 3,000 × *g* for 5 min. Cells were resuspended in 1 mL aggregation buffer or 10% ASM and diluted to OD_600_ ~0.5–1.0 in 1 mL in polyurethane cuvettes. Cuvettes were incubated at room temperature without agitation, and the OD_600_ was measured at regular intervals up to 180 min.

For the aggregate disruption assays using the different enzymes, aggregates were disrupted by adding a concentration range of each of the enzymes to the aggregation buffer and incubating at room temperature with mixing for 1 h. The recombinant hydrolases PslG, PelA, Sph3, Ega3, and Dispersin B were purified using nickel affinity chromatography as described previously (64, 66–69). His-tagged SIR_1591^28–468^, lacking the first 27 residues including its putative transmembrane domain, was recombinantly expressed in *E. coli* and purified to homogeneity (Table S1). Purification was performed using nickel affinity chromatography as previously described (64). Chitinase, cellulase, and Proteinase K were purchased from Sigma Aldrich, and DNase I was from Biobasic. After incubation with the enzymes for 1 h, the cuvettes were further left standing at room temperature, and aggregation assaye wasd as described above. The OD 600 over time at different concentrations of the enzymes was analyzed using Prism and the EC_50_ values calculated as previously described (64).

### Scanning electron microscopy

Overnight cultures were centrifuged at 3,000 × *g* for 5 min. Cells were washed in 1 mL PBS and centrifuged at 3,000 × *g* for 5 min. Cells were resuspended in 1 mL PBS and diluted to OD_600_ ~1.0. Bacterial samples were prepared for analysis by scanning electron microscopy (SEM) using critical point drying and gold sputtering as previously described (16). Briefly, bacteria were transferred onto coverslips and fixed with 2.5% glutaraldehyde. The coverslips were washed with PBS and dehydrated using a series of ethanol washes at 50%, 70%, 90%, and 100% ethanol for 15 min each. Samples were then critical point dried by replacing the ethanol with CO_2_ and sputter coated in gold. The sample processing was completed by the Nanoscale Biomedical Imaging Facility at The Hospital of Sick Children (Toronto, Ontario), and the images were obtained using a Scanning Electron Microscope (FEI 30Kv voltage model XL-30 SEM with a tungsten gun and secondary electron detector) at The Hospital for Sick Children.

### Generation of GalNAc-specific monoclonal antibody

The synthesis of the GalNAc trisaccharide used for antibody generation is outlined in Scheme S1, and the details of the methods and materials used can be found in the Supplemental information (70, 71). The GalNAc trimer was conjugated to bovine serum albumin (BSA) as described previously (72). Mice were immunized with a BSA-(GalNAc)_3_ glycoconjugate followed by natural infection with *Aspergillus fumigatus* Af293 to further mature the immune response. For the immunization protocol, mice were injected with 500 µL of vaccine preparation containing 10 µg of glycoconjugate (BSA-GalNAc3) and 50 µg of Alum via two administration pathways: 300 µL was injected intraperitoneally, and 200 µL was injected sub-cutaneously. At days 21 and 42, 100 µL of the vaccine preparation was administered intravenously as a boost. On day 51, mice were challenged with *A. fumigatus* contained in agar beads at half the dose reported previously (73). At day 65, a last vaccine boost was administered intravenously. On day 69, mouse spleens were harvested for the preparation of hybridomas by an external company (MediMabs). Mice sera were screened for the production of antibodies directed against the oligosaccharide, and immortalized hybridomas were created from their splenocytes. Monoclonal antibody candidates were then screened for reactivity against BSA-(GalNAc)_3_. To identify clones that specifically recognize the GalNAc found in the *P. aeruginosa* Pel and *A. fumigatus* galactosaminogalactan (GAG) exopolysaccharides, Pel- and GAG-sufficient and -deficient culture supernatants of *P. aeruginosa* PA14 and *A. fumigatus* Af293 were used.

### Slot blot analysis

Slot blots using the α-(GalNAc)_3_ antibody were performed as follows. Cells were harvested by centrifugation (4,000 × *g* for 5 min) from 5 mL of *S. intermedius* cultures grown overnight at 37°C in THY broth. Cell were resuspended in 1 mL aggregation buffer at an optimized OD of 2.0 and centrifuged at 4,000 × *g* for 5 min. The supernatant was discarded, and the cell pellets were resuspended in 100 µL of 0.5 M EDTA, pH 8.0. Cells were boiled for 20 min with occasional vortexing, and centrifuged (16,000 × *g* for 10 min) to harvest the supernatant containing cell-associated Pel. Cell-associated Pel was treated with proteinase K (final concentration, 0.5 mg/mL) for 60 min at 60°C, followed by 30 min at 80°C to inactivate proteinase K. For the immunoblots, 20 µL of cell-associated Pel, prepared as described above, was pipetted onto a nitrocellulose membrane using a vacuum slot blot apparatus (Schleicher & Schuell SRC072/0 Minifold II Slot-Blot System). The membrane was blocked with 5% (wt/vol) skim milk in Tris-buffered saline with Tween-20 (TBS-T) for 1 h at room temperature and probed with adsorbed α-(GalNAc)_3_ at a 1:1,000 dilution in 1% (wt/vol) skim milk in TBS-T overnight at 4°C with shaking. Blots were washed three times for 5 min each with TBS-T, probed with goat α-rabbit HRP-conjugated secondary antibody (Bio-Rad) at a 1:2,000 dilution in TBS-T for 45 min at room temperature with shaking, and washed again. All immunoblots were developed using SuperSignal West Pico (Thermo Scientific) following the manufacturer’s recommendations. For protein loading controls, 100 µL of the OD normalized cells were lysed in a lysis buffer (20 mM Tris-HCl, 2 mM EDTA, 1.2% Triton, 20 mg/mL lysozyme). SDS loading buffer was added to the lysates and boiled at 100°C for 5 min. Lysates were then run on an SDS-PAGE gel and stained with coomassie blue to visualize the total protein content.

### Confocal imaging

For all confocal imaging studies, bacteria were grown either on poly lysine-coated coverslips or in eight-well chamber slides (Nunc Lab-Tek Chambered Coverglass) for ~22 h at 37C in a CO_2_ incubator. Bacteria were fixed on the coverslip or the chambered slides using 4% paraformaldehyde after carefully removing the media. Bacteria were stained using the α-(GalNAc)_3_ antibody (1:1,000 dilution of a 1 mg/mL solution) after blocking using a Carboblock solution (Biolynx Inc). The slides were washed 3× with PBS post-primary and stained with an anti-mouse Alexa fluor 488 secondary antibody for 1 h followed by DAPI staining for 5 min. Bacteria were then washed 3× with PBS. Coverslips were mounted and chamber slides were directly imaged with PBS using a Spinning disk confocal microscope(Imaging Facility, Hospital for Sick Children). Images were processed using Volocity software.

### *In vivo* murine skin infection model

Six- to 8-week-old female BALB/c mice were obtained from Charles River Laboratories and utilized for establishing the skin abscess model. Prior to infection, the mice were anesthetized with isoflurane, and their dorsal backs were shaved. The mice were subsequently intradermally inoculated with *S. intermedius* C1365 wild-type or Δ*pelF* strains in 100 µL of PBS. Bacteria for infection were grown overnight in THY broth as described above. On the day of infection, the OD_600_ was determined, and cultures were diluted to obtain 10^8^ bacteria/100 µL of PBS. Mice were carefully monitored for any signs of skin infection. On day 6, the mice were euthanized, and the abscesses were measured, excised and their weights recorded. Abscesses from 5 mice in each group were homogenized and dilutions plated for enumerating the bacterial burden on THY plates. The plates were incubated overnight at 37°C in a 5% CO_2_ incubator and the CFU/abscess was calculated the next day. All data points were plotted using GraphPad Prism, and an unpaired *t* test was used for statistical analysis.

### PBMC isolation and cytokine quantification

Peripheral blood mononuclear cells (PBMCs) from four male donors were recovered from heparinized blood via density gradient centrifugation using Ficoll-Plaque separation media (GE Healthcare) and Leucosep tubes (Greiner Bio-One). PBMCs frozen at −120°C in DMSO and heat-inactivated AB serum (Corning) were quickly thawed, washed, and seeded to a density of 1 × 10^5^ cells/well in 200 µL of RPMI-1640 supplemented with 10% fetal bovine serum (FBS; Gibco), 100 U/mL penicillin G, 100 µg/mL streptomycin, and 2 mM L-glutamine using round-bottom 96-well plates (Corning). Following an overnight rest period at 37°C in atmospheric conditions supplemented with 5% CO_2_, PBMCs were stimulated using heat-killed bacteria (10 min at 75°C) at a multiplicity of infection (MOI) of 1:1 or with 10 ng/mL of LPS (Invivogen) for 24 h at 37°C in the presence of 5% CO_2_. Cells were subsequently centrifuged at 1,500 × *g* for 5 min, and the supernatants were recovered and stored at −20°C until further use. PBMCs cytokine profiles were obtained with the Human Cytokine Array Proinflammatory Focused High Sensitivity T-cell discovery array 13-plex cytokine profiling kit (Eve Technologies) measuring GM-CSF, IFNγ, IL-1β, IL-2, IL-4, IL-5, IL-6, IL-8, IL-10, IL-12p70, IL-13, MCP-1, and TNF-α. Triplicates were pooled from individual PBMC stimulation experiments and cytokines quantification was performed once. Cytokine production was normalized with the LPS-PBMC control in order to correct for donor and experimental heterogeneity.

## RESULTS

### *S. intermedius* clinical strains have variable surface adherent biofilm phenotypes

**Fig 2:**
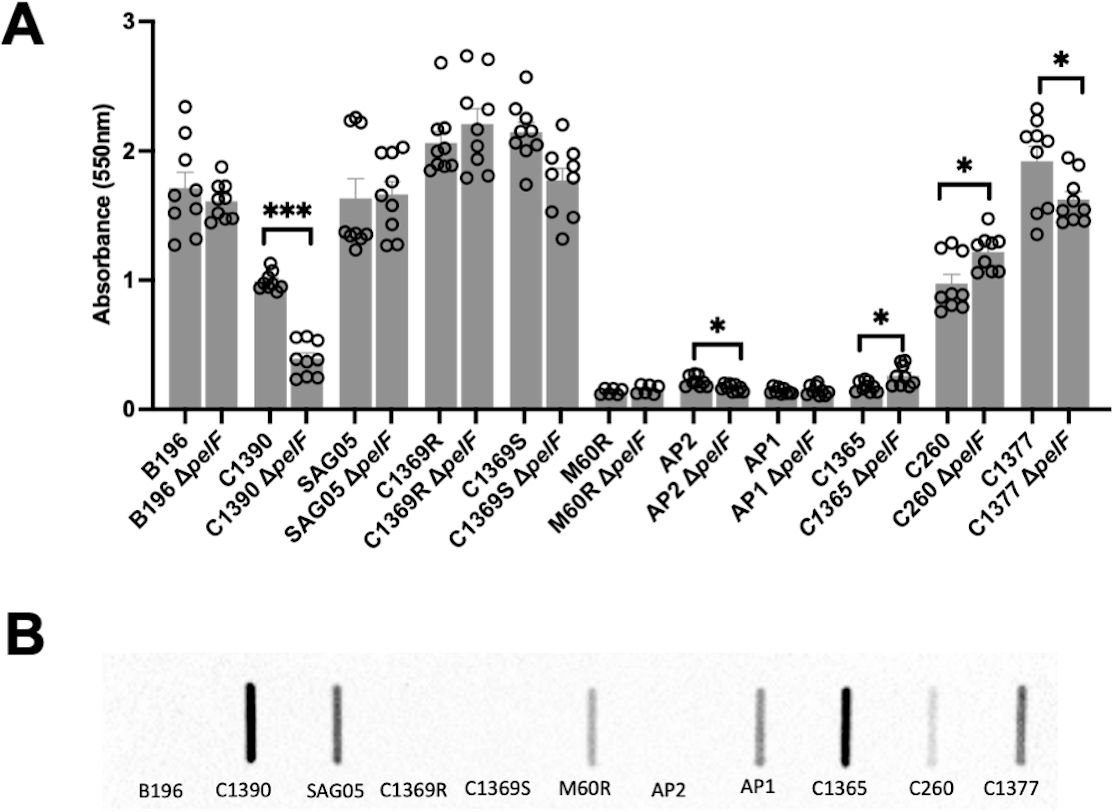
Biofilm biomass does not correlate with Pel production. (**A**) Eleven *S. intermedius* clinical strains and their corresponding Δ*pelF* mutants were assayed using crystal violet staining after being grown in THY broth on poly-lysine coated polystyrene multi-well plates (Cellstar) for 24 h at 37°C in 5% CO_2_. The crystal violet assays were performed three times. The Δ*pelF* mutants were compared with their corresponding wild-type strain using an unpaired *t* test. * =*P* < .05, **=*P* < .01, ***=*P* < .005. (**B**) Presence of Pel- like exopolysaccharide was detected in the 11 clinical strains using a slot blot. Cells were harvested and treated with proteinase-K to remove protein before probing for the presence of Pel using a monoclonal antibody raised against a BSA-(GalNAc)_3_ glycoconjugate followed by a secondary anti-mouse IgM conjugated to horseradish peroxidase (HRP).

To determine whether the *pel* operon plays a role in biofilm formation in *S. intermedius*, we utilized a collection of 11 clinical isolates from different anatomical sites and geographical locations (Table 1) and tested their ability to adhere to poly-lysine-coated polystyrene multi-well plates using a crystal violet adherence assay (Fig. 2A). We found significant variability in the capacity of the different *S. intermedius* strains to form adherent biofilms under the conditions tested. Some strains, B196, C1390, SAG05, C1369R, C1369S, C260, and C1377, consistently produced high levels of biofilm biomass after 24 h, whereas others, such as M60R, AP1, AP2, and C1365, adhered poorly, resulting in low recoverable biofilm biomass. To determine whether the levels of biofilm biomass observed were dependent on the Pel polysaccharide, we used a slot-blot assay and our specific monoclonal antibody raised against a trimer of *N*-acetylgalactosamine (α-(GalNAc)_3_ mAb) to assess production of Pel (Fig. 2B). We found no correlation between Pel production and the location that the strain was isolated from (Table 1) or whether the strain adhered strongly to the plate. B196, C1365R, and C1369S adhere to the plate but do not produce Pel, whereas C1390, SAG05, C260, and C1377 adhere and make Pel (Fig. 2A and B). Similarly, we found no correlation between the non-adherent strains and Pel production, as we observed Pel production in the M60R, AP1, and C1365 strains but not AP2. The absence or presence of Pel in the biofilm was confirmed by live cell confocal microscopy of selected strains, B196, C1365, C1369R, and C1377, grown on glass (Fig. S1). We also constructed *pelF* deletion mutants (Δ*pelF*) and tested these strains using our crystal violet adherence assay (Fig. 2A). As PelF is the glycosyltransferase, *pelF* deletion mutants lack the ability to synthesize the Pel polymer. As anticipated, we found no change in the adherence phenotype of the B196, C1369S, and C1369R *pelF* deletion mutants, consistent with their lack of Pel production (Fig. 2). Interestingly, two strongly adhering strains, C1377 and C1390, did show significant differences when *pelF* was deleted, but these strains still adhered significantly better than the non-adherent M60R, AP1, AP2, and C1365 strains (Fig. 2A). In contrast, deletion of *pelF* in C260 led to a statistically significant increase in adherence, whereas there was no change in phenotype in the Δ*pelF* SAG05 strain relative to wild-type. Deletion of *pelF* in the non-surface adherent strains did not alter this phenotype. Collectively, these data suggest that the role of Pel in the production of adherent biofilms is highly variable between strains under the conditions tested and suggests that other matrix components such as eDNA or proteins may also influence the formation of adherent biofilms.

### Auto-aggregation in *S. intermedius* is dependent on Pel biosynthesis

**Fig 3:**
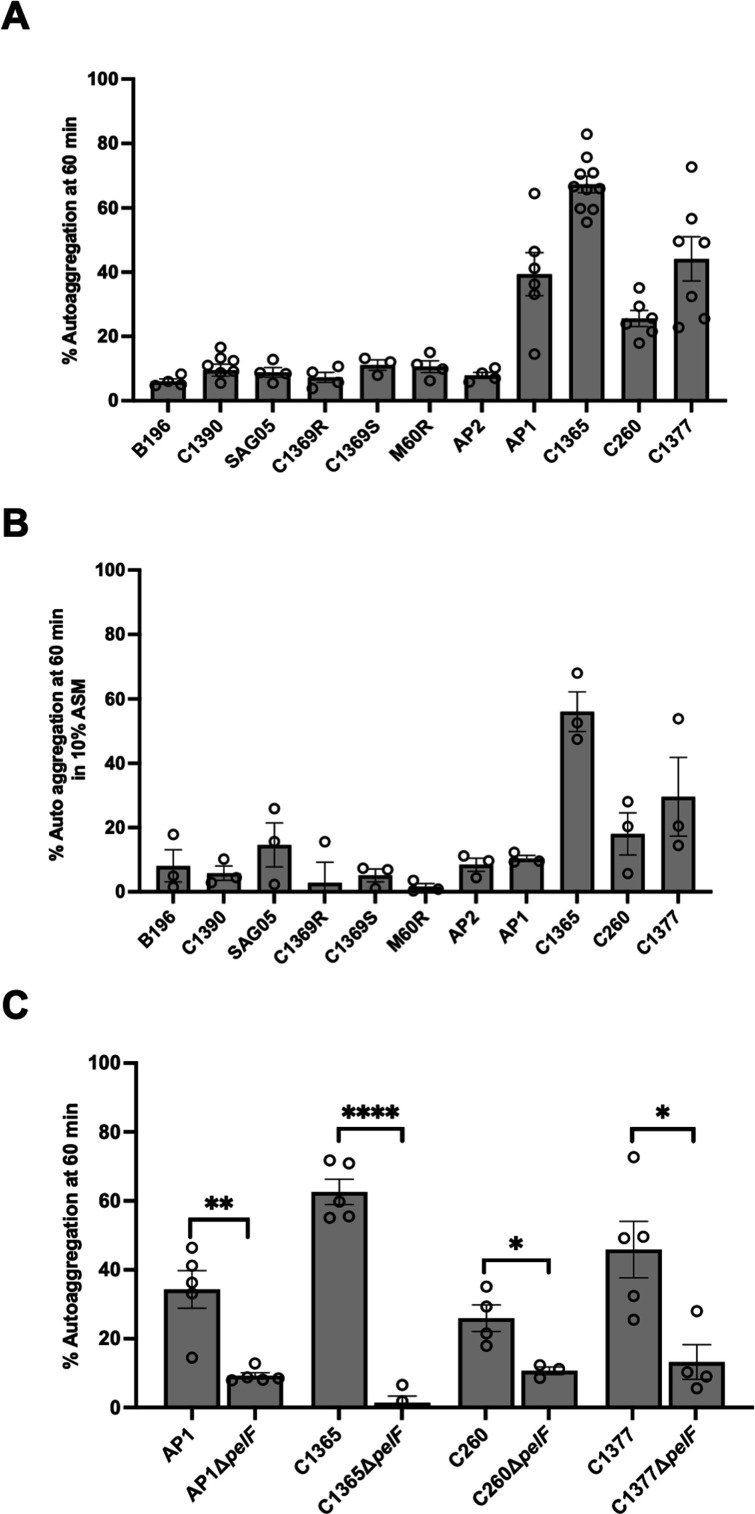
Pel biosynthesis contributes to auto-aggregation in hyper-aggregating strains of *S. intermedius.* (**A**) *S. intermedius* clinical strains were screened for auto-aggregation capacity at 60 min in an aggregation buffer. Four hyper-aggregating strains were identified. (**B**) Auto-aggregation of *S. intermedius* clinical strains was tested in 10% artificial sputum medium (ASM); 10% ASM was prepared by diluting ASM in phosphate-buffered saline (PBS). (**C**) Hyper-aggregation was abrogated by deletion of the glycosyl transferase *pelF* (Δ*pelF*) in the AP1, C1365, C260, and C1377 strains, indicating that the process is dependent on Pel biosynthesis. Aggregation assays were performed at least three times. Aggregates formed by each wild-type strain were compared with the corresponding Δ*pelF* mutant using an unpaired *t* test. * =*P* < .05, **=*P* < .01, ***=*P* < .005, ****=*P* < .001

Bacterial aggregates have been linked to a variety of chronic infections and aggregation is known to be an important driver of biofilm adhesion and cohesion in several bacterial species (8, 74). As the levels of biofilm biomass measured in our crystal violet adherence assay did not correlate with Pel production, we next screened the clinical isolates for their ability to auto-aggregate in suspension using an aggregation buffer. We found four strains, AP1, C1365, C260, and C1377, that displayed high levels of auto-aggregation over time in the conditions tested (Fig. 3A & Fig. S2A). Interestingly, the degree of auto-aggregation in these strains correlates with the amount of Pel produced (Fig. 2B and 3A). Although all the aggregating strains make Pel (Fig. 2B), there is no obvious correlation between non-aggregating strains and Pel production, as C1390, SAG05, and M60R make Pel, but B196, C1369R, and C1369S do not (Fig. 2B and 3A). To ensure that the aggregation phenotype observed was not due to the buffer conditions used, we repeated the assay in a more physiologically relevant medium, 10% artificial sputum medium (ASM). The results of this assay are consistent with those observed in our aggregation buffer (Fig. 3B). To determine whether Pel was involved in the hyper-aggregation phenotype, we examined aggregation in the AP1, C1365, C260, and C1377 *pelF* mutants. For each Δ*pelF* mutant, we found a significant reduction in aggregation relative to the parental strain, suggesting a role for Pel in this phenotype (Fig. 3C).

**Fig 4:**
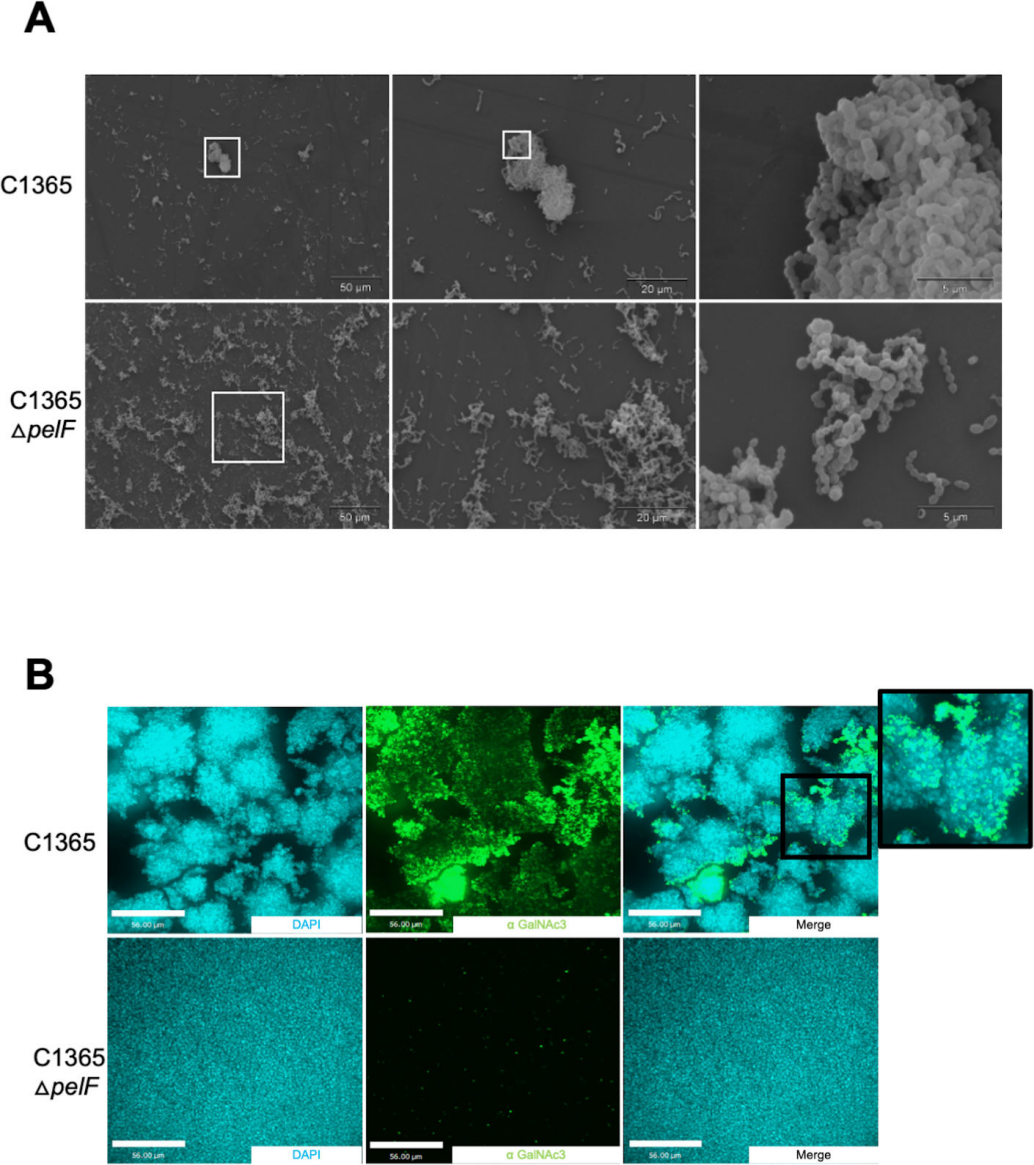
Pel is a major component of the biofilm matrix in a hyper-aggregating strain of *S. intermedius.* (**A**) Scanning electron microscopy demonstrated that clumping of the hyper-aggregating strain *S. intermedius* C1365 is reduced dramatically when *pelF* is deleted. The micrographs represent each of the strains under three different magnifications. (**B**) Confocal images of *S. intermedius* C1365 wild-type and Δ*pelF* deletion strain. Immunostaining was performed using a primary mouse anti-(GalNAc)_3_ antibody followed by secondary anti-mouse Alexafluor488 (Green) and DAPI to stain for bacterial cells (Blue). Images were collected using a Spinning Disk Confocal Microscope and processed using Volocity.

Scanning electron microscopy (SEM) of the hyper-aggregating C1365 strain corroborated our aggregation assay results, as we found that the wild-type C1365 strain formed large, tightly p..acked cell aggregates with very few distinguishable chains of streptococci, whereas the cells in the C1365 Δ*pelF* strain were much more dispersed, forming much smaller, more loosely packed aggregates, and chains of streptci were clearly visible (Fig. 4A). As the SEM analysis of C1365 aggregates did not reveal a visible biofilm matrix, confocal microscopy was performed using a fluorescently labeled version of the α-(GalNAc)_3_ mAb to verify that a Pel-like polymer was produced and determine its cellular location within the aggregates. Comparison of the C1365 wild-type and Δ*pelF* mutant clearly reveals localization of the α-(GalNAc)_3_ mAb to the aggregates formed by the wild-type strain. No aggregates and no signal for the α-(GalNAc)_3_ mAb werwasserved in the Δ*pelF* mutant (Fig. 4B and Fig. S2B). Combined, these data suggest that *S. intermedius* strains that aggregate require the production of the Pel polymer for this phenotype.

### Phenotypic differences in AP1 and AP2 strains are the result of a *pelD* mutation

Two of the clinical *S. intermedius* strains (AP1 and AP2) were isolated from the pleural fluid of a patient with pneumonia. These strains displayed different phenotypes in our adherence and aggregation assays. Although neither strain formed surface-adherent biofilms (Fig. 2A), AP1 aggregated, and this aggregation was dependent on the presence of *pelF* (Fig. 3B). Further characterization of these strains also revealed two different colony morphotypes. When grown on Congo-red agar plates, AP1 colonies wrinkled around the circumference, whereas AP2 had a smoother surface (Fig. S3A). Slot blot analysis also reveals that although AP1 makes Pel, AP2 does not. Scanning electron micrographs of these two strains showed that although AP1 forms aggregates, AP2 in the absence of Pel is more diffuse with the bacterial chains more separated (Fig. S3B). To investigate the genetic basis of these phenotypic differences, we performed whole genome sequencing on the two strains. The sequencing revealed that the strains are isogenic except for three single nucleotide polymorphisms (SNPs). The first SNP is in the coding region of *glmU,* which encodes an *N*-acetylglucosamine-1-phosphate uridyltransferase that synthesizes UDP-*N*-acetylglucosamine (UDP-GlcNAc) (54). Although the mutation results in a glycine-to-serine substitution at residue 304, *glmU* is an essential gene, and therefore, the function of this protein is unlikely to be disrupted. The second SNP is located 35 nucleotide base pairs upstream of a gene involved in copper homeostasis, *cutC*. The third SNP is found in the *pelD* coding region. Although the *pelD* gene is intact in AP1, the SNP in AP2 introduces a premature stop codon in the coding region of the third transmembrane helix of PelD. The premature stop would truncate the protein at residue 304, resulting in a protein that would lack the C-terminal GAF domain. Given our finding that deletion of *pelF* impacts aggregation in AP1 (Fig. 3B), we hypothesized that the *pelD* SNP in AP2 may result in the non-aggregation phenotype observed in this strain. *pelD* deletion mutants were therefore constructed in both AP1 and AP2, and their auto-aggregation phenotypes were assessed. Time-course experiments show that AP2 begins aggregating as quickly as 15 min in an aggregation buffer (Fig. S2A). Deletion of *pelD* in AP1 resulted in a significant decrease in auto-aggregation at 15 min relative to the wild-type parental strain, although there was no detectable phenotypic change in the AP2 Δ*pelD* mutant (Fig. S3C). Taken together, these data suggest that Pel production is required for the auto-aggregation observed in AP1 and that the SNP identified in AP2 reduces auto-aggregation to levels equivalent to that of the AP1 *pelD* deletion mutant.

### Pel production in *S. intermedius* C1365 requires *pelDEA_DA_FG* but not *SIR_1591-1594*

**Fig 5:**
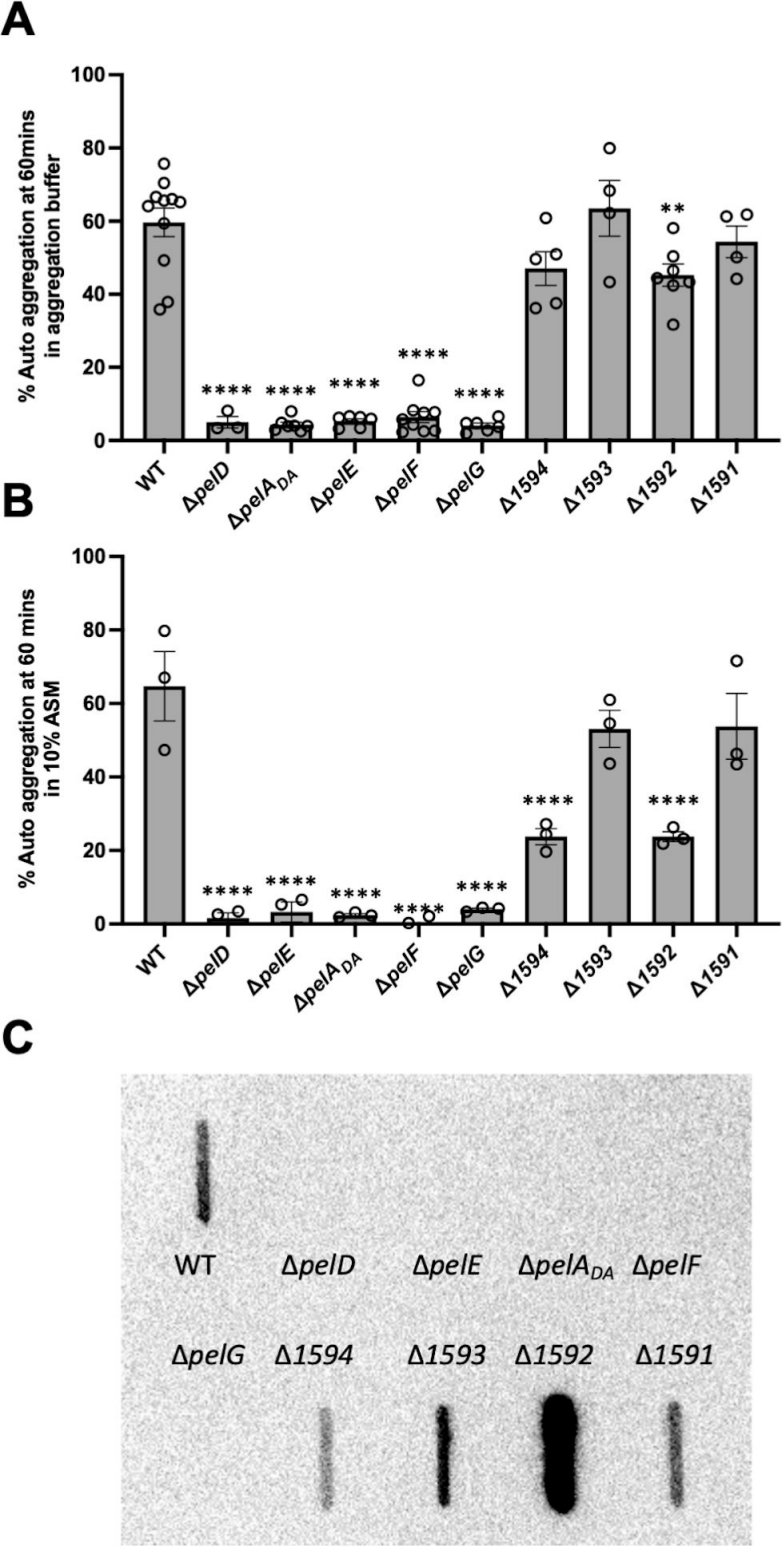
Contribution of *S. intermedius pel* genes to auto-aggregation. Each of the genes in the *S. intermedius* C1365 *pel* gene cluster was deleted by allelic replacement, and the deletion strains were screened for auto-aggregation capacity in (**A**) an aggregation buffer or (**B**) 10% artificial sputum medium (ASM). Statistical analyses were performed using a one-way ANOVA followed by a Dunnett’s multiple comparisons test comparing each of the mutants with the wild-type. **P < 0.01, ****P < 0.0001. (**C**) *S. intermedius* C1365 wild-type and *pel*-deletion strains were assayed for the presence of a Pel-like exopolysaccharide by slot blot. Cells were harvested and treated with proteinase K to remove protein before probing for the presence of a Pel-like polysaccharide using a monoclonal antibody raised against a BSA-(GalNAc)_3_ glycoconjugate followed by a secondary anti-mouse IgM conjugated to HRP.

Having demonstrated that both *pelD* and *pelF* are important for the aggregation phenotype, we next assessed the role of the rest of the genes in the *pel* operon in the hyper-aggregating C1365 strain, including the four additional non-canonical *pel* genes. Similar to the results obtained for the Δ*pelD* and Δ*pelF* mutants, the deletion of each of *pelA_DA_, pelE*, and *pelG* resulted in a loss of the aggregation phenotype both in our aggregation buffer and in 10% ASM (Fig. 5A and B). Growth curves demonstrate that there were no growth defects in any of these mutants (Fig. S4). Although the mutants appear to grow better than the wild-type in these experiments, this is likely due to the aggregation phenotype of the wild-type strain, which would affect the accuracy of the OD readings. We also performed whole genome sequencing using Nanopore technology of both the wild-type and the Δ*pelF* mutant to ensure that there were no polar effects on downstream genes. One SNP was detected in a gene encoding a predicted serine protease causing an amino acid change from proline to serine. Aggregation was observed in both aggregation buffer and 10% ASM for each of the *SIR_1591, SIR_1592, SIR_1593,* and *SIR_1594* deletion mutants (Fig. 5A and B). In a theggregation buffer, we observed a small decrease in aggregation in the Δ*SIR_1592* and Δ*SIR_1594* mutants relative to wild-type, which was significant for Δ*SIR_1592*. However, in 10% ASM, these two strains exhibited a > 50% decrease in aggregation (Fig. 5B). To determine whether deletion of genes in the operon affected Pel production, a slot-blot analysis was performed using our α-(GalNAc)_3_ mAb. This analysis clearly demonstrates that in the strains that aggregate, that is, wild-type and each of the *SIR_1591, SIR_1592, SIR_1593*, and *SIR_1594* mutants, Pel is present in the cell pellets (Fig. 5C). As observed previously for *P. aeruginosa* and *B. cereus,* deletion of any of *pelDEA_DA_FG* results in the loss of Pel signal. Given the equal amounts of protein present in our normalized samples (Fig. S5), our slot blot results suggest that the levels of Pel produced in the Δ*SIR_1592* and Δ*SIR_1594* mutants vary significantly from wild-type, with the Δ*SIR_1592* mutant producing more Pel and Δ*SIR_1594* producing less Pel (Fig. 5C). Taken together, these results suggest that each of the genes in the canonical *pelDEA_DA_FG* operon operonsquired for the production of a Pel-like GalNAc-rich polymer that drives aggregation in *S. intermedius*. The changes in the levels of Pel produced and the reduced aggregation observed in the *SIR_1592* and *SIR_1594* mutants also suggest that these genes are important for these processes. Our analyses of *SIR_1592* and *SIR_1594* suggest that *SIR_1594* encodes a secreted protein whose N-terminal region (residues 62-472) has significant homology (22% identity) to *B. subtilis* and *B. cereus* CotH, whereas *SIR_1592* is predicted to encode a transmembrane protein with four TM segments (residues 22–143) and an intracellular C-terminal aspartate kinase, chorismate mutase, and TyrA (ACT) domain (Fig. S6).

### SIR_1591 is an active glycosyl hydrolase that can prevent C1365 aggregation

**Fig 6:**
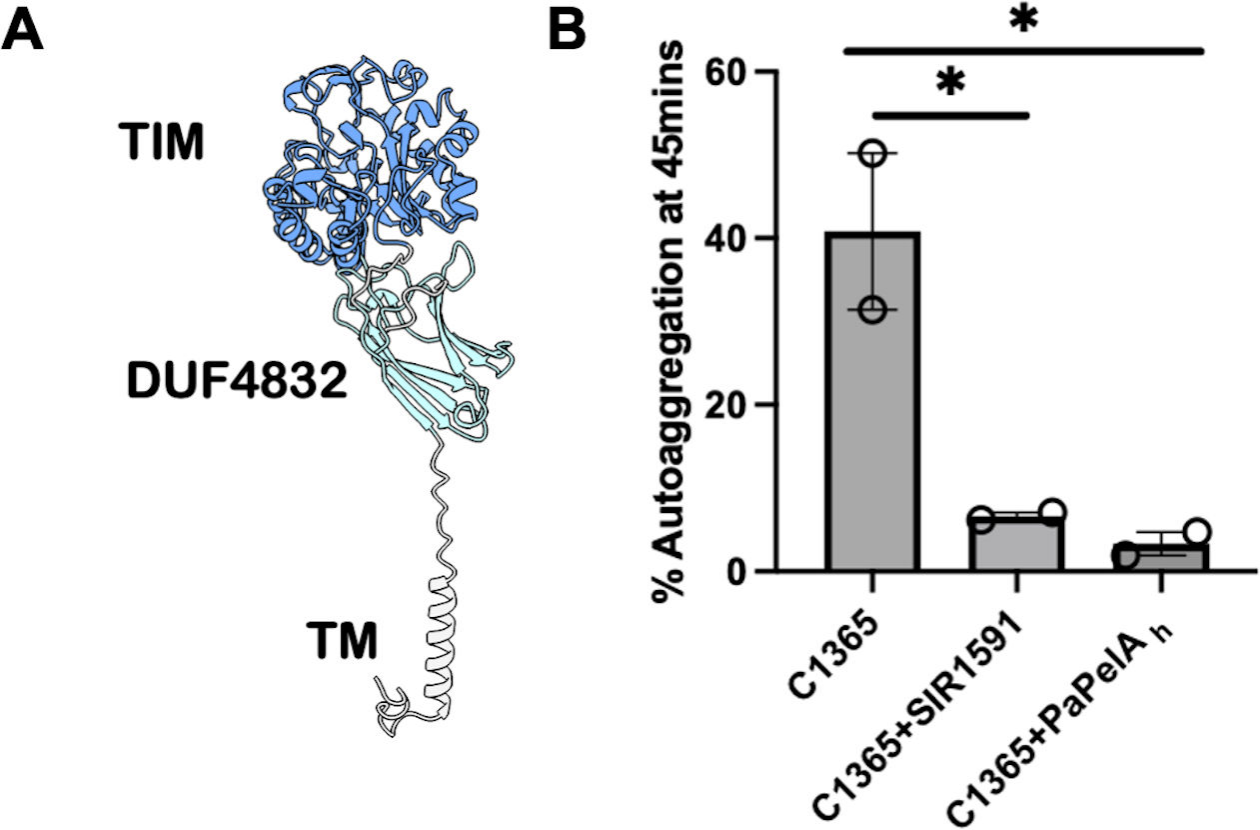
*S. intermedius* SIR_1591 is a functional glycoside hydrolase. (**A**) AF2 model of SIR_1591 showing the transmembrane, DUF4832, and TIM barrel domains of the protein. Dark and light blue represent the N- and C-terminal domains of the protein. (**B**) Recombinantly purified SIR_1591 disrupts preformed aggregates of *S. intermedius* strain C1365 when added exogenously. The activity of this enzyme is comparable with *P*aPelA_H_. Aggregation was determined by measuring OD 600 nm over time. The aggregates were completely disrupted by 45 min when incubated with 1 µM of SIR_1591 and *Pa*PelA_H_. Statistical analysis was performed using a one-way ANOVA and Dunnett’s multiple comparisons test. *P < 0.05.

Although our previous results suggested that SIR_1591 does not play a role in aggregation or Pel production, we were nonetheless intrigued by this gene since it is predicted to be a glycoside hydrolase. Glycoside hydrolases are a common feature of exopolysaccharide biosynthetic systems (75), and in *P. aeruginosa,* the hydrolase activity of PelA has been associated with both production of cell-free Pel, as well as matrix disruption and dispersal (19, 76). Our bioinformatics analyses suggest that SIR_1591 is found on the extracellular surface of the bacterium, in keeping with the identification of a homolog of this protein in the secretome of *Streptococcus gordonii (77*) and the location of the mature Pel polymer. Examination of the AF2-predicted structure available on UniProt suggests that SIR_1591 is a two-domain protein with residues 60–350 forming a TIM barrel domain that the DALI and Foldseek servers (78, 79) suggest has structural similarity to beta-amylases and beta-galactosidases (Fig. 6A). The AF2 model also suggests that residues 1-28 form a transmembrane helix, whereas residues 44–52 and 351–454 form a seven β-strand domain of unknown function (DUF4832). Homologs of this domain have been found at the C-terminus of many glycoside hydrolases and have structural similarities to carbohydrate binding domains (80). To determine whether *SIR_1591* is indeed a glycoside hydrolase, we recombinantly expressed SIR_1591 minus its transmembrane helix (SIR_1591^28-468^) with a His-tag in *Escherichia coli* and purified the protein to homogeneity. We found that the exogenous addition of this protein or the hydrolase domain of *P. aeruginosa* PelA, PelA_H_, both disrupted pre-formed aggregates of C1365 (Fig. 6B). These data suggest that like other *pel* operons, *SIR_1591* encodes an active glycoside hydrolase, and that the polymer made by *S. intermedius* is similar to Pel produced by *P. aeruginosa*.

### *S. intermedius* Pel is composed predominantly of GalNAc

**Fig 7:**
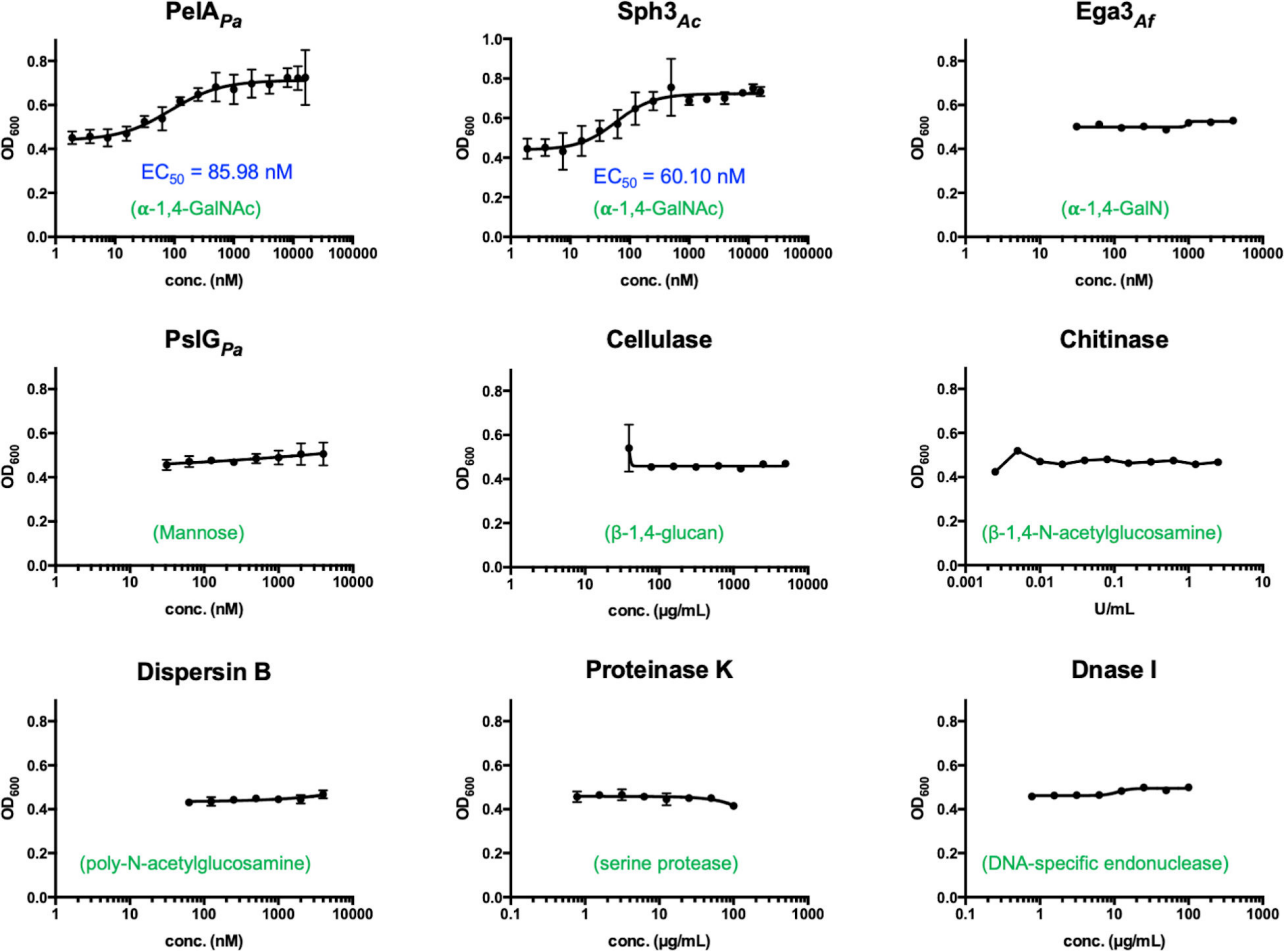
*S. intermedius* C1365 aggregation is driven by an exopolysaccharide chemically similar to *P. aeruginosa* Pel**.** Disruption assays were performed on *S. intermedius* C1365 aggregates, using a panel of enzymes targeting different matrix components. The graphs indicate dose-response curves to examine the disruption of aggregation by the exogenous treatment of each enzyme. Enzymes were added at a range of concentrations to bacterial aggregates and OD600 was measured over time. EC_50_ values were calculated using nonlinear least-squares fitting to a dose-response model using GraphPad Prism. Error bars indicate SEM.

To further characterize the Pel-like polysaccharide produced by *S. intermedius* C1365 aggregates, enzymatic disruption assays were performed. Cell aggregates were treated with enzymes that target specific biofilm matrix components and have been shown to disrupt biofilm aggregates in other bacterial species (64, 66, 81–84). Using a panel of nine enzymes, including glycan-specific, protein-specific, and DNA-specific enzymes, we found that although enzymes that target DNA- or protein-specific components of the matrix did not disrupt the aggregates, the hydrolase domains of *P. aeruginosa* PelA, PelA_H_, and *Aspergillus clavatus* Sph3 were able to disrupt *S. intermedius* C1365 aggregates (Fig. 7). These enzymes disrupted the aggregates with EC_50_ values in the nanomolar range. Although both PelA_H_ and Sph3 have been characterized as α−1,4-*N*-acetylgalactosaminidases, PelA_H_ can disrupt *P. aeruginosa* Pel-dependent biofilms, but Sph3 cannot (66). Differences in their active site architectures result in distinct enzyme-substrate interactions, particularly with respect to their ability to hydrolyze deacetylated stretches within polymers. Although neither enzyme can hydrolyze GalN oligosaccharides, PelA_H_ can hydrolyze partially deacetylated substrates better than Sph3. Of note, *Aspergillus fumigatus* Ega3*,* an endo-acting α−1,4-galactosaminidase that can readily disrupt *P. aeruginosa* biofilms (81), did not disrupt the *S. intermedius* aggregates. These data provide further evidence that the hyper-aggregation observed in *S. intermedius* C1365 is dependent on an α−1,4-*N*-acetylgalactosamine rich, Pel-like polysaccharide, but also suggests that there may be differences in the degree of de-*N-*acetylation of the polymer relative to *P. aeruginosa* or *B. cereus* (67, 81). The ability of Sph3 but not Ega3 to disrupt the aggregates suggests that *S. intermedius* Pel is predominantly composed of GalNAc.

### Pel-dependent aggregation leads to longer-lasting infections *in vivo*

**Fig 8:**
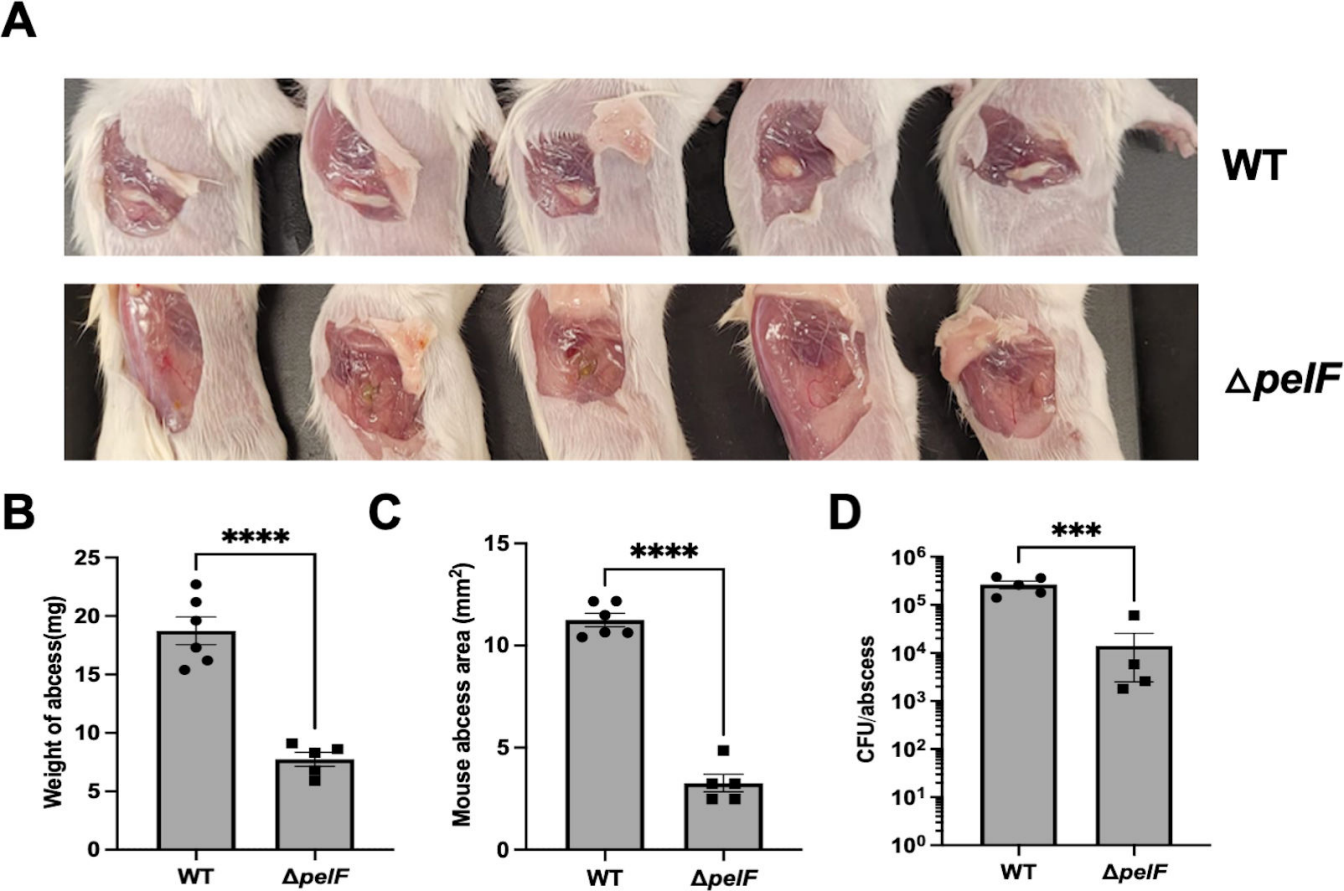
Lack of Pel allows for faster clearance of *S. intermedius* infections in a mouse abscess model. (**A**) Female BALB/c mice were injected intradermally with 1 × 10^8^ CFU of *S. intermedius* C1365 and the corresponding Δ*pelF* mutant. Infection was followed over 6 days for the development of abscesses. Mice were monitored daily and sacrificed at day 6 post-infection. Weight of the abscess (**B**) and the abscess area (**C**) were measured post-sacrifice and excision. Abscess homogenates were serially diluted and plated on THY agar. Plates were incubated for 24 h at 37°C with 5% CO_2_ and CFU/abscess quantified. As observed in (**D**), the CFUs recovered from the mutant were significantly lower than from abscesses infected with WT *S. intermedius*. All statistical analyses were performed using unpaired *t* tests on GraphPad Prism. ****=*P* < .001, ***=*P* < .005

Previous studies in *P. aeruginosa* have shown that the absence of cell-free Pel enhances virulence in two infection models (19). Use of Pel- and Psl-specific hydrolases in combination with antibiotics reduces bacterial burden in both a lung and wound model of *P. aeruginosa* infection, underlining the importance of the formation of biofilm aggregates and the role of exopolysaccharides in infection (67, 68, 85). To establish the importance of Pel and aggregate formation in *S. intermedius* virulence, we performed animal studies using a murine subcutaneous abscess model (86). Female BALB/c mice were infected with either the wild-type C1365 strain or the corresponding Δ*pelF* mutant intradermally and the infection was followed over 6 days. At the end of 6 days, the abscess size was measured, and the excised abscesses were weighed before homogenization and plating for bacterial burden. Although we observed visible abscesses in mice infected with the wild-type strain, the mutant group had significantly smaller abscesses (Fig. 8; Fig. S6). We found that one of the six mice in the mutant group had completely resolved the infection with no visible abscess after six days. The weight (Fig. 8B) and size (Fig. 8C) of the abscesses were significantly lower in mice infected with the Pel-deficient strain than in the wild-type-infected mice, indicating that aggregates persisted for a longer time locally at the site of infection. We hypothesize that non-aggregating bacteria dispersed systemically and were rapidly cleared by the immune system. This hypothesis is supported by the significantly reduced bacterial burden recovered from the abscesses of mice infected with the Δ*pelF* mutant (Fig. 8D).

### *S. intermedius* aggregation affects host immune responses

**Fig 9:**
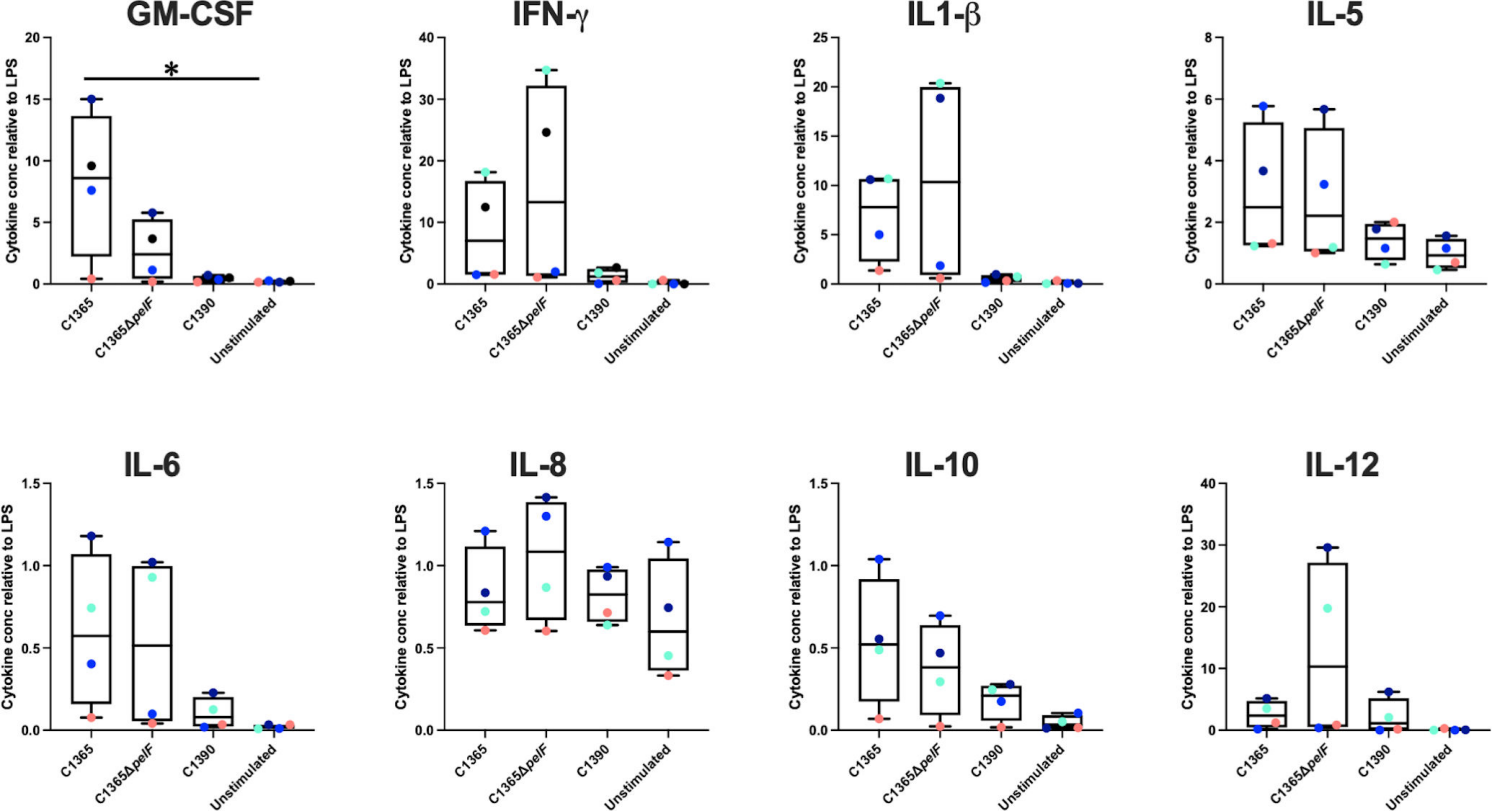
Hyperaggregating strains of *S. intermedius* elicit a distinct GM-CSF and IL-12 response in human PBMCs. Heat-killed *S. intermedius* cells were incubated with primary human PBMCs isolated from four different donors, indicated by the colored circles. Cytokine levels were normalized to LPS-induced controls to account for differences in the immunogenicity of the donors. US = unstimulated control. Only the GM-CSF response to C1365 WT showed a significant difference compared with the unstimulated control. Statistical analysis was performed using one-way ANOVA and Tukey’s multiple comparisons test. *P < 0.05.

Results from the murine abscess model indicate that Pel-dependent aggregation is important for the virulence of *S. intermedius* and that these aggregates are not cleared by the immune system as efficiently as the non-aggregating *pelF* mutant. Induction of the immune response by bacteria is dependent on a myriad of factors. Predominantly, it is initiated when pattern-recognition receptors sense conserved motifs on the bacterial surface or the surface of the aggregates. This could include different components such as teichoic acids, lipopolysaccharides, surface proteins, or the exopolysaccharides present on the surface of the aggregates. We hence hypothesized that the expression of extracellular polysaccharides such as Pel would enable the bacteria to evade the innate immune response and alter cytokine responses. To test this, peripheral blood mononuclear cells (PBMCs) were isolated from four healthy donors and incubated with heat-killed *S. intermedius* cells *in vitro*. The cytokine profile of these PBMCs was then analyzed by ELISA and compared with controls. We found that the average levels of IL-6, IL-5, IL-8, IL-10, GM-CSF, IL-1β, IFNγ, and IL-12 cytokines were increased in the hyper-aggregating C1365 strain compared with the non-aggregating C1390 strain, although these differences did not reach statistical significance (Fig. 9). Although the effects observed for the C1365 Δ*pelF* mutant were highly variable between donors and no changes reached statistical significance, a reduction in the level of GM-CSF induction was observed in PBMCs exposed to *S. intermedius* C1365 Δ*pelF* relative to the levels induced by exposure to the wild-type strain. Levels of IL-12 were also reduced in the wild-type strain compared to the Δ*pelF* mutant. These data support the hypothesis that Pel affects how *S. intermedius* interacts with the host immune system. Further studies are needed to characterize the exact mechanisms by which the changes in cytokine levels affect the immune response and thereby clearance of the bacteria from infection sites.

## DISCUSSION

Herein, we show that clinical strains of *S. intermedius* can auto-aggregate and that this aggregation is dependent on a Pel-like GalNAc-rich polysaccharide produced by *pelDEA_DA_FG* (Fig. 1–4). Interestingly*,* we found that surface-adherent biofilm formation was largely independent of Pel production (Fig. 2A). Animal studies using an abscess model show that Pel-dependent aggregation leads to longer-lasting infections *in vivo* (Fig. 8)*,* and although highly variable, cytokine assays suggest that the response of some host immune cells may be different when Pel is absent (Fig. 9). Although the current study focused on the Pel dependent aggregating strains of *S. intermedius,* our analyses of eleven different clinical isolates enables us to define four different phenotypes based on their ability to form surface adherent biofilms and/or aggregates. The phenotypes observed were: (i) aggregating and surface adherent (C1377 and C260); (ii) aggregating and non-surface adherent (C1365 and AP1); (iii) non-aggregating and surface adherent (B196, SAG05, C1369S, C1369R, and C1390); and (iv) non-aggregating and non-surface adherent (M60R and AP2). Although our data clearly support a role for Pel in aggregation, it also suggests a limited role for Pel in surface adherent biofilm formation. These data contrast with observations for *P. aeruginosa* and *B. cereus* ATCC 10987, where Pel plays a role in adherent biofilm formation in these species (16, 21). The limited role of Pel in *S. intermedius* adherence could be related to the degree of deacetylation, and therefore charge, of the mature Pel polymer. Our aggregate disruption assays revealed that the α−1,4-*N-*acetylgalactosaminidases PelA and Sph3, but not the α−1,4-galactosaminidase Ega3, disrupt *S. intermedius* aggregates. These data suggest that *S. intermedius* Pel may be composed predominantly of GalNAc, which would lead to a neutral rather than cationic polymer. Differences in the charge of the polymer will have a profound effect on its function. The structure of *S. intermedius* Pel and what other factor(s) in the biofilm matrix of these clinical strains contribute to the different phenotypes, especially adherence, remains to be determined.

Previous analyses of the *pel* operon in different bacterial species (15–17) identified *pelD* as a key difference between the *S. intermedius* and *P. aeruginosa* and *B. cereus pel* operons. PelD has been classified into four groups based on the presence or absence of the GGDEF, SDR, or degenerate SDR domains. *S. intermedius* PelD falls into class IV, as it contains a degenerate SDR domain and lacks the GGDEF domain; hence, it does not have the ability to bind c-di-GMP (17). Bioinformatic analyses of available SMG genome sequences also indicate that these species do not encode homologs to any known diguanylate cyclase enzymes. This suggests that unlike *P. aeruginosa* Pel biosynthesis, which is regulated at the transcriptional and post-translational levels *via* binding of c-di-GMP to FleQ and PelD, respectively (33, 34, 87), transcription of the *pel* operon and activation of the *S. intermedius* Pel biosynthetic complex must be regulated by different mechanisms. Previous gene cluster analyses revealed *pel* operons across multiple SMG species, providing strong evidence to suggest that this operon plays an important role in the biology of these species, not just *S. intermedius,* and that *pelD* is a functional part of the system (Fig. 5 & S3) (17). The importance of PelD in auto-aggregation is clearly demonstrated by the AP1 and AP2 clinical isolates (Fig. 5 and S3). Although the aggregating behavior may be beneficial for the bacteria, the presence of the non-aggregating AP2 morphotype within the pleural fluid is interesting. It is established that changes within the environment are a major determinant of genetic diversity. Consequently, it is possible that the transition of the bacterial strain from the initial site of infection to the pleural cavity where the isolates were recovered, or the dynamic microenvironment-induced empyema, could have selected a specific bacterial phenotype potentially more adapted to its new niche. How Pel biosynthesis is regulated in the absence of c-di-GMP is yet to be determined. However, c-di-AMP and ppGpp are potential candidates as, in other gram-positive species, these molecules play a role in regulating the response to nutrient limitation and biofilm formation (88–92). Given the multiple levels of Pel regulation in gram-negative bacteria, it will be interesting to study how, in the absence of c-di-GMP, other factors regulate the *pel* operon and its expression in *Streptococci*.

The other notable difference when comparing the *S. intermedius pel* operon with the operons found in *P. aeruginosa* or *B. cereus* is the presence of the four additional genes, *SIR_1594-SIR_1591*. Our results show that the deletion of *SIR_1592* and *SIR_1594* significantly altered the levels of Pel produced and the auto-aggregation propensity in ASM (Fig. 5). The lack of phenotypic change in the glycoside hydrolase *SIR_1591* mutant (Fig. 5 and 6) was unanticipated as deletion of the hydrolase adjacent to the *pel* operon in *B. cereus* (*pelA_H_*) and abrogation of hydrolase activity in *P. aeruginosa* PelA lead to an increase in biofilm formation (16, 19). Despite the absence of a phenotype for the *SIR_1591* mutant, we found that SIR_1591 is an active glycoside hydrolase (Fig. 6) ,suggesting that SIR_1591, like *P. aeruginosa* PelA, could play a role in biofilm dispersal (76). Although the mechanisms by which *SIR_1592* and *SIR_1594* contribute to Pel-dependent aggregation also remain to be determined, it is interesting to note that these proteins ,as well as SIR_1593 ,may play a role in phosphate metabolism (Fig. S6B). Bioinformatics analyses suggest that SIR_1594 is a secreted protein with an N-terminal serine kinase CotH domain and C-terminal protein-protein interaction fibronectin type 3 (FN3) domain (Fig. S7). CotH has been implicated in the regulation of spore coat formation in *Bacillus subtilis* and *B. cereus (93, 94*), but as *Streptococci* do not sporulate, we hypothesize that if SIR_1594 is an active serine kinase, its activity has been re-purposed in this species. Both SIR_1592 and SIR_1593 also contain domains that have been implicated in phosphate metabolism. SIR_1592 has a MgtC transmembrane domain and an intracellular C-terminal aspartate kinase, chorismate mutase, and TyrA (ACT) domain (Fig. 1 ; Fig. S7). The MgtC domain of *Salmonella enterica* inhibits its own ATP-synthase and deregulates phosphate metabolism by targeting PhoR, thus promoting intra-macrophage survival and long-term virulence (95). Finally, SIR_1593 is predicted to contain a polyphosphate (polyP) polymerase domain. PolyP is an anionic polymer with a diverse array of functions in bacteria including phosphate storage, stress resistance, biofilm formation, and virulence (96). Bacterial polyphosphates have also been associated with better survival in human macrophages (97). The relevance of *SIR_1592, SIR_1593*, and *SIR_1594* in Pel-dependent aggregation and/or virulence is also underscored by our analyses that fail to find this syntenic arrangement of these three genes outside of the Pel locus. Given that functional homologs of *SIR_1592, SIR_1593*, and *SIR_1594* play major roles in host-pathogen interactions and stress responses in bacteria (98–100), future experiments will need to focus on examining the role of these genes in the context of the host using, for example, epithelial or macrophage cell lines.

Our cytokine assays suggest that host immune cells may respond differently when Pel is absent (Fig. 9). Elevated levels of GM-CSF were detected from donor PBMCs when exposed to the wild-type C1365 Pel-producing and -aggregating strain. These GM-CSF levels were different from the non-aggregating C1365 Δ*pelF* mutant and the non-aggregating C1390 *S. intermedius* strain. GM-CSF is an immune-modulating cytokine that plays a critical role in maintaining the pulmonary immune system and is known to drive the immune functions of alveolar macrophages (101, 102). GM-CSF-negative mice have also been shown to clear Group B Streptococcal infections slower than wild-type mice (102). Although elevated levels of GM-CSF would indicate an increase in the number of infiltrating macrophages, it has been shown previously that the beneficial impacts of bacterial aggregation are more pronounced for larger aggregates, as they are more resistant to phagocytosis (103). Thus, an elevated immune response to aggregates could lead to more harm than good by increasing inflammation levels. Levels of the cytokine IL-12 also showed differences in PBMCs from two donors. Moreover, the PBMC response to specific strains was donor-dependent, suggesting both strain and host variability contribute to the virulence potential of this group of bacteria (104). This heterogeneity within the host response across strains has also been previously observed in *S. aureus* and *S. pyogenes* (105, 106). The IL-12 response in aggregating strains was muted compared with the response in the Δ*pelF* strains. IL-12 levels are critical in early infection control, generation, and maintenance of an adaptive immune response and phagocytosis (107). These results may be indicative of the ability of the host immune response to more effectively clear Pel-deficient, non-aggregating strains. Although we did not observe any significant trends in other cytokine levels, this study only assessed the role of PBMCs. Future studies will explore whether other aspects of the immune system control the host response to infection.

In summary, this study establishes a role for Pel in aggregation and infection in SMG strains, specifically *S. intermedius*. We show that Pel leads to persistent infections *in vivo* and can modulate host immune responses. Our analysis of the operon highlights differences between Streptococcal spp. and other gram-positive and -negative species such as *B. cereus* and *P. aeruginosa*. Future studies will focus on determining the c-di-GMP-independent mechanism of regulation of Pel in these bacteria and the precise role the four additional genes play in this process.

## Data Availability

All data generated or analyzed during this current study are included in this published article and its supporting information. Genomic data for AP1 and AP2 have been deposited in GenBank: SAMN41984811 and SAMN41989511, respectively.

## SUPPLEMENTAL MATERIAL

Supplemental material mbio.01196-25-s0001.pdf Supplemental text, figures, and tables.

ASM does not own the copyrights to Supplemental Material that may be linked to, or accessed through, an article. The authors have granted ASM a non-exclusive, world-wide license to publish the Supplemental Material files. Please contact the corresponding author directly for reuse.
