# Supplementary material for: Auto-aggregation in Streptococcus intermedius is driven by the Pel polysaccharide
Source: mBio. 2025 Jul 7;16(8):e01196-25. doi: 10.1128/mbio.01196-25 (PMC12345266; doi:10.1128/mbio.01196-25)
Supplement: Supplemental material — Supplemental text, figures, and tables. [file mbio.01196-25-s0001.pdf]

Supplemental Information

Auto aggregation in *Streptococcus intermedius* is driven by the Pel polysaccharide

Deepa Raju[a,*], Siobhán A. Turner[a,*], Karla Castro[a,b,#], Gregory B. Whitfield[a,b,#], Daphnée Lamarche[c,#], Sahil Mahajan[d], Roland Pfoh[a], Stephanie H.W. Chuang[a,b], François Le Mauff[e,f,g], Maju Joe[h], Susmita Sarkar[h], Todd L. Lowary[h,i], Donald C Sheppard[e,f,g], Daniel J. Wozniak[d], Michael G. Surette[c], P. Lynne Howell[a,b,%].

[a]Program in Molecular Medicine, The Hospital for Sick Children, Toronto, Ontario, Canada

[b]Department of Biochemistry, University of Toronto, Toronto, Ontario, Canada

[c]Department of Biochemistry and Biomedical Sciences and the Michael G. DeGroote Institute for Infectious Disease Research, McMaster University, Hamilton, Ontario, Canada.

[d]Department of Microbiology, The Ohio State University, Columbus, OH, 43210, USA

[e]Department of Microbiology and Immunology, and Medicine, McGill University, Montreal, Québec, Canada

[f]Department of Medicine, McGill University, Montreal, Canada

[g]McGill Interdisciplinary Initiative in Infection and Immunity, Montreal, Canada

[h]Department of Chemistry, University of Alberta, Alberta, Canada

[i]Institute of Biological Chemistry, Academia Sinica, and Institute of Biochemical Sciences, National Taiwan University, Taipei, Taiwan

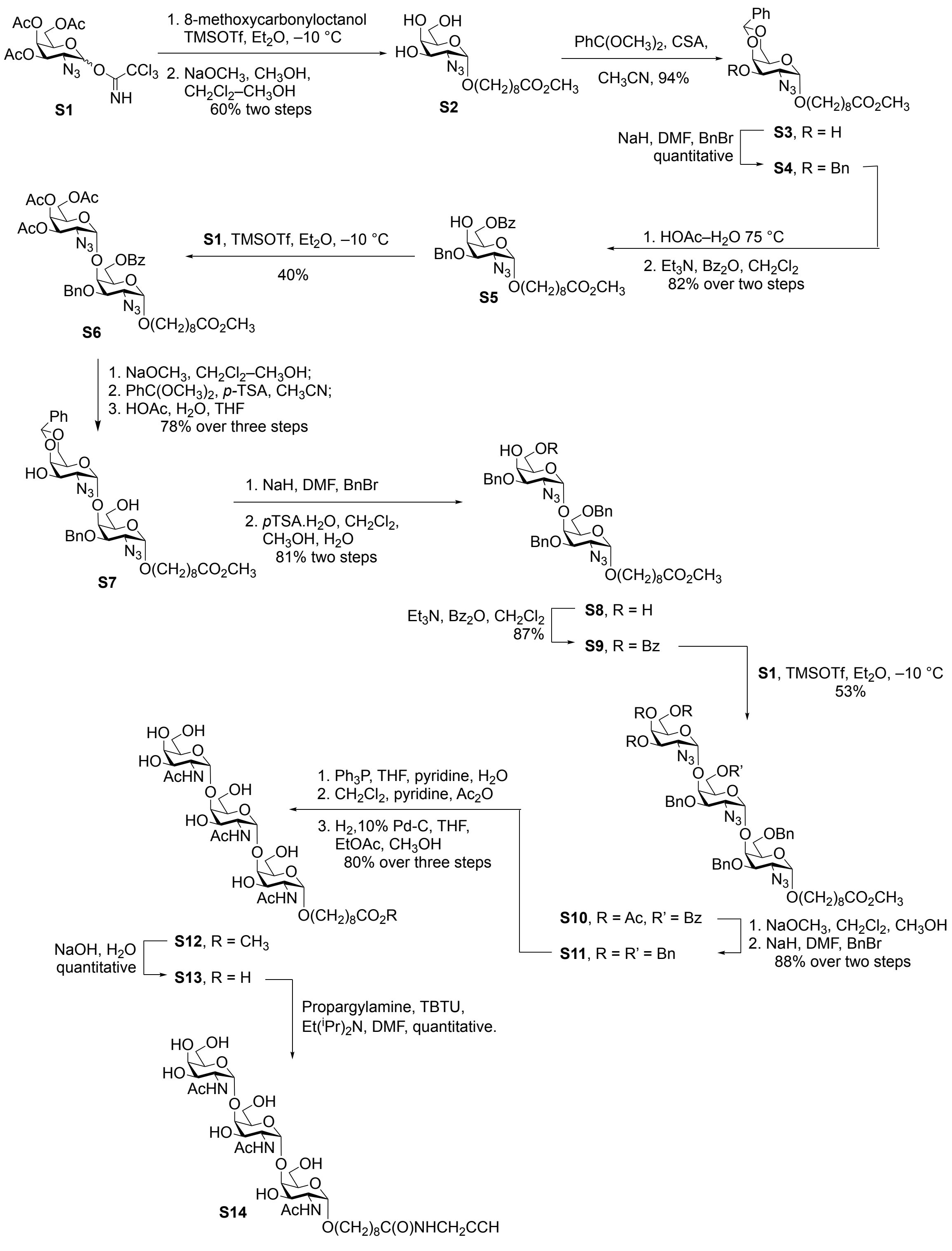

Scheme S1: Synthesis of GalNAc trisaccharide **S14** for antibody generation.

Supplemental Materials & Methods

Synthesis of GalNAc trimer for antibody generation

Reactions were carried out in oven-dried glassware. All reagents were purchased from commercial sources and were used without further purification unless noted. Reaction solvents were purified by successive passage through columns of alumina and copper under nitrogen. Unless stated otherwise, all reactions were carried out at room temperature under a positive pressure of argon and were monitored by TLC on Silica Gel 60 F_{254} (0.25 mm, E. Merck). Spots were detected under UV light or by charring with acidified *p*-anisaldehyde solution in EtOH. Unless otherwise indicated, all column chromatography was performed on Silica Gel (40–60 μM). The ratio between silica gel and crude product ranged from 100 to 50:1 (w/w). Optical rotations were measured at 22±2 °C. ^{1}H NMR spectra were recorded at 700 MHz or 500 MHz and chemical shifts were referenced to either TMS (0.0, $CDCl_3$) or CD_3OD (3.30, CD_3OD) or HOD (4.78, D_2O). ^{1}H data were reported as though they were first order. ^{13}C NMR (APT) spectra were recorded at 176 MHz or 125 MHz, and ^{13}C chemical shifts were referenced to internal $CDCl_3$ (77.23, $CDCl_3$), or CD_3OD (48.9, CD_3OD) or external acetone (31.07, D_2O). Organic solutions were concentrated under vacuum at <40 °C. Electrospray mass spectra were recorded on samples suspended in mixtures of THF with CH_3OH and added NaCl.

8-Methoxycarbonyloctyl 2-azido-2-deoxy-α-D-galactopyranoside (S2). A mixture of trichloroacetimidate **S1**[1] (prepared from 5.92 g, 17.87 mmol of the hemiacetal), 8-methoxycarbonyl octanol[2] (4.2 g, 22.3 mmol) and molecular sieves (4Å, 1.27 g) in dry diethyl ether (115 mL) was stirred under argon for 30 min., cooled to −15 °C and then TMSOTf (20 μL) was added dropwise. The stirring continued at −15 °C for about 10 min and then allowed to warm to −10 °C over the next 25 min before the acid was quenched by the addition of a few drops of triethylamine. The mixture was filtered through a pad of Celite and washed with CH_2Cl_2 (50 mL). The combined filtrate was concentrated, and the residue was purified by column chromatography (4:1, *n*-hexane–EtOAc) to afford the product as a thick syrup (α:β ratio 2:1); R_f 0.27 (3:1, *n*-hexane–EtOAc); HRMS (ESI) calcd. for $(M+Na)^+$ $C_{22}H_{35}NaN_3O_{10}$ 524.2215, found 524.2205. To a solution of the above compound (α/β mixture) in CH_2Cl_2–CH_3OH (7:2, 90 mL) was added catalytic sodium methoxide in CH_3OH to bring the pH of the reaction mixture to 8–9. After stirring for 48 h, the reaction mixture was neutralized by the addition of pre-washed Amberlite IR 120 H^+ resin. The solution was filtered and the filtrate was concentrated to a syrupy residue that was purified by chromatography (1:4, *n*-hexane–EtOAc) to give **S2** (4.0 g, pure α product, 60% over two steps; the β product was not isolated) as a white foam; R_f 0.28 (1:4, *n*-hexane–EtOAc), ^{1}H NMR (500 MHz, $CDCl_3$, δ_H); δ 4.93 (d, J = 3.5 Hz, 1H, H-1), 4.08–3.99 (m, 2H), 3.90–3.77 (m, 3H), 3.70–3.66 (m, 1H), 3.66 (s, 3H, OCH_3, included in multiplet), 3.48–3.41 (m, 2H), 2.30 (t, J = 7.5 Hz, 2H), 2.26 (br. s, 2 × OH), 1.63–1.55 (m, 4H), 1.38–1.27 (m, 8H); ^{13}C NMR (125 MHz, $CDCl_3$, δ_C) 174.70 (C=O), 98.26 (C-1), 77.33, 77.08, 76.82, 70.23, 70.11, 69.56, 68.46, 68.02, 67.91, 62.56, 62.43, 60.19, 51.52, 49.99, 49.82, 49.65, 49.48, 49.31, 49.14, 48.97, 34.08, 29.31, 29.08, 29.06, 28.98, 26.00, 24.85; HRMS (ESI) calcd. for $(M+Na)^+$ $C_{16}H_{29}NaN_3O_7$ 398.1898, found 398.1891.

8-Methoxycarbonyloctyl 4,6-*O*-benzylidene-2-azido-2-deoxy-α-D-galactopyranoside (S3). To a solution of **S2** (3.99 g, 10.62 mmol) in dry acetonitrile (50 mL) was added benzaldehyde dimethylacetal (2.0 mL, 13.33 mmol) followed by camphorsulfonic acid (CSA, 120 mg). The reaction mixture was stirred for 5 h before THF (10 mL), water (1.0 mL) and HOAc (0.5 mL) were added in succession. After stirring for 30 min, the solution was neutralized by the addition of triethylamine and concentrated to a syrupy residue that was purified by column chromatography (4:1, *n*-hexane–EtOAc) to afford **S3** (4.61 g, 94%) as a thick syrup; R_f 0.25 (3:1, *n*-hexane–EtOAc); $[\alpha]_D$ +143.26 (c 0.7, $CHCl_3$); ^{1}H NMR (500 MHz, $CDCl_3$, δ_H) 7.54–7.48 (m, 2H), 7.45–7.37 (m, 3H), 5.60 (s, 1H, PhCH), 5.02 (d, J = 3.3 Hz, 1H,

H-1), 4.34–4.27 (m, 2H), 4.21 (ddd, J = 10.7, 3.7 Hz, 1H), 4.12 (dd, J = 12.6, 1.8 Hz, 1H), 3.78–3.70 (m, 2H), 3.69 (s, 3H, OCH_3), 3.59–3.48 (m, 2H), 2.51 (d, J = 10.7 Hz, 1H), 2.33 (t, J = 7.5 Hz, 2H), 1.69–1.58 (m, 4H), 1.43–1.31 (m, 8H); ^{13}C NMR (125 MHz, $CDCl_3$, δ_C) 174.33 (C=O), 137.39, 129.36, 128.34, 126.26, 101.32, 98.70 (C-1), 77.32, 77.07, 76.81, 75.63, 69.33, 68.76, 67.39, 62.80, 62.68, 60.71, 51.49, 34.09, 29.40, 29.16, 29.15, 29.06, 26.05, 24.92; HRMS (ESI) calcd. for $(M+Na)^+$ $C_{23}H_{33}NaN_3O_7$ 486.2211, found 486.2212.

8-Methoxycarbonyloctyl 4,6-*O*-benzylidene-3-*O*-benzyl-2-azido-2-deoxy-α-D-galactopyranoside (S4). To a solution of **S3** (0.28 g, 0.32 mmol) in dry DMF (3 mL) cooled to 0 °C, sodium hydride (60% dispersion in mineral oil, 40 mg, 1.0 mmol) was added in portions followed by benzyl bromide (0.1 mL, 0.85 mmol) dropwise. The reaction mixture was then allowed to warm to room temperature and stirred for 2 h before CH_3OH (0.1 mL) was added dropwise at 0 °C. The solution was diluted with CH_2Cl_2 (10 mL) and washed with brine (1 mL) and water (5 mL). The CH_2Cl_2 layer was separated, dried with anhydrous Na_2SO_4 and concentrated to a syrupy residue that was purified by chromatography (85:15, *n*-hexane–EtOAc) to afford **S4** (0.33 g, quantitative) as a thick syrup; R_f 0.46 (3:1, *n*-hexane–EtOAc); ^{1}H NMR (500 MHz, $CDCl_3$, δ_H) 7.58–7.52 (m, 2H), 7.47–7.43 (m, 2H), 7.41–7.29 (m, 6H), 5.50 (s, 1H, PhC*H*), 5.02 (d, J = 3.4 Hz, 1H, H-1), 4.83–4.73 (m, 2H), 4.29–4.24 (m, 2H), 4.09–4.02 (m, 2H), 3.91 (dd, J = 10.6, 3.5 Hz, 1H), 3.75–3.64 (m, 5H), 3.52 (dt, J = 9.8, 6.6 Hz, 1H), 2.34 (t, J = 7.5 Hz, 2H), 1.69–1.58 (m, 4H), 1.41–1.30 (m, 8H); ^{13}C NMR (125 MHz, $CDCl_3$, δ_C) 174.30 (C=O), 138.02, 137.69, 128.98, 128.45, 128.19, 127.87, 127.79, 126.24, 100.95 (Ph-*C*H), 98.59 (C-1), 77.32, 77.07, 76.81, 74.48, 73.19, 71.29, 69.47, 68.67, 62.84, 58.79, 51.48, 34.10, 29.73, 29.40, 29.18, 29.08, 26.03, 24.94.

8-Methoxycarbonyloctyl 6-*O*-benzoyl-3-*O*-benzyl-2-azido-2-deoxy-α-D-galactopyranoside (S5). A solution of **S4** (0.33 g, mmol) in HOAc–H_2O (4:1, 30 mL) was heated at 75 °C for 2.5 h, cooled to room temperature and then concentrated to obtain a syrupy residue that was dried overnight under vacuum. To a solution of this crude diol in CH_2Cl_2 (30 mL) was added triethylamine (1.0 mL, 7.1 mmol), followed by benzoic anhydride (0.4 g, 1.76 mmol) and the solution was stirred for 36 h. The reaction mixture was then concentrated and the residue was purified by column chromatography (4:1, *n*-hexane–EtOAc) to afford **S5** (0.28 g, 82% over two steps) as a thick syrup; R_f 0.29 (4:1, *n*-hexane–EtOAc), ^{1}H NMR (500 MHz, $CDCl_3$, δ_H) 8.09–8.03 (m, 2H), 7.64–7.57 (m, 1H), 7.50–7.31 (m, 7H), 4.96 (d, J = 3.6 Hz, 1H), 4.80 (d, J = 11.3 Hz, 1H), 4.73 (d, J = 11.3 Hz, 1H), 4.62 (dd, J = 11.5, 4.8 Hz, 1H), 4.52 (dd, J = 11.5, 7.6 Hz, 1H), 4.13–4.10 (m, 2H), 3.97 (dd, J = 10.4, 3.1 Hz, 1H), 3.74–3.64 (m, 5H), 3.49 (dt, J = 9.7, 6.7 Hz, 1H), 2.31 (t, J = 7.6 Hz, 2H), 1.66–1.54 (m, 4H), 1.34–1.22 (m, 8H); ^{13}C NMR (125 MHz, $CDCl_3$, δ_C) 174.29 (C=O), 166.34 (C=O), 137.19, 133.21, 129.90, 129.68, 128.74, 128.44, 128.36, 128.29, 128.07, 97.90 (C-1), 77.30, 77.05, 76.80, 76.09, 72.30, 68.51, 67.94, 66.53, 63.99, 59.06, 51.47, 34.09, 29.73, 29.33, 29.15, 29.11, 29.04, 26.02, 24.93.

8-Methoxycarbonyloctyl 3,4,6-tri-*O*-acetyl-2-azido-2-deoxy-α-D-galactopyranosyl-(1→4)-6-*O*-benzoyl-3-*O*-benzyl-2-azido-2-deoxy-α-D-galactopyranoside (S6). A mixture of trichloroacetimidate **S1**[1] (prepared from 0.64 g, 1.9 mmol of the hemiacetal), acceptor **S5** (0.61 g, 1.1 mmol) and molecular sieves (4Å, 0.69 g) in dry CH_2Cl_2–Et_2O (1:1, 36 mL) was stirred under argon for 30 min., cooled to −15 °C and then TMSOTf (35 μL) was added dropwise. The mixture was stirred initially at −10 °C for 20 min and then warmed to 8 °C over the next 1 h before the acid was quenched by the addition of a few drops of triethylamine. The mixture was filtered through a pad of Celite and washed with CH_2Cl_2 (30 mL). The combined filtrate was concentrated and the residue was purified by chromatography (3:1, *n*-hexane–EtOAc) to afford **S6** (0.38 g, 40%) as a white foam; R_f 0.3, (3:1, *n*-hexane–EtOAc, two runs); $[\alpha]_D$ +114.71 (*c* 0.3, $CHCl_3$); ^{1}H NMR (700 MHz, $CDCl_3$, δ_H) 8.02–7.98 (m, 2H), 7.59–7.20 (m, 8H), 5.39 (dd, J = 3.2, 1.5 Hz, 1H), 5.34 (dd, J = 11.1, 3.2 Hz, 1H), 5.05 (d, J = 3.6 Hz, 1H, H-1), 4.98 (d, J

= 3.6 Hz, 1H, H-1), 4.77 (d, *J* = 11.8 Hz, 1H), 4.73 (d, *J* = 11.8 Hz, 1H), 4.64–4.55 (m, 3H), 4.17 (d, *J* = 2.8 Hz, 1H), 4.08 (t, *J* = 7.0 Hz, 1H), 3.94–3.87 (m, 2H), 3.81 (dd, *J* = 11.1, 3.6 Hz, 1H), 3.70 (dd, *J* = 10.8, 3.6 Hz, 1H), 3.65–3.61 (m, 1H), 3.63 (s, 3H, OCH_3, included in multiplet), 3.52–3.43 (m, 2H), 2.26 (t, *J* = 7.6 Hz, 2H), 2.08 (s, 3H, $COCH_3$), 2.03 (s, 3H, $COCH_3$), 1.84 (s, 3H, $COCH_3$), 1.60–1.52 (m, 4H), 1.28–1.18 (m, 8H); ^{13}C NMR (176 MHz, $CDCl_3$, δ_C) 174.22 (C=O), 170.02 (C=O), 169.93 (C=O), 169.69 (C=O), 165.90 (C=O), 137.09, 133.37, 129.61, 129.56, 128.52, 127.92, 127.46, 99.10 (C-1), 97.93 (C-1), 77.17, 76.99, 76.81, 75.44, 74.89, 72.68, 68.66, 68.59, 68.42, 67.13, 66.69, 62.51, 60.43, 59.54, 58.27, 51.40, 34.02, 29.27, 29.08, 29.05, 28.98, 25.94, 24.87, 20.63, 20.57, 20.50; HRMS (ESI) calcd. for $(M+Na)^+$ $C_{42}H_{54}NaN_6O_{15}$ 905.3539, found 905.3542.

8-Methoxycarbonyloctyl 4,6-*O*-benzylidene-2-azido-2-deoxy-α-D-galactopyranosyl-(1→4)-3-*O*-benzyl-2-azido-2-deoxy-α-D-galactopyranoside (S7). To a solution of compound **S6** (0.375 g, 0.42 mmol) in CH_2Cl_2–CH_3OH (1:1, 16 mL) was added sodium methoxide in CH_3OH to bring the pH of the reaction mixture to 8–9. The solution was stirred overnight and then neutralized by the addition of pre-washed Amberlite IR 120 H^+ resin, filtered and the filtrate was concentrated to a syrupy residue that was dried under high vacuum overnight; R_f 0.07 (2:3, *n*-hexane–EtOAc). To a solution of this compound (0.26 g, 0.4 mmol) in dry acetonitrile (12 mL) was added benzaldehyde dimethylacetal (0.4 mL, 2.7 mmol) followed by anhydrous *p*-toluenesulfonic acid (TSA, 17 mg). The reaction mixture was stirred for 24 h before THF (5 mL), water (0.5 mL) and HOAc (0.1 mL) were successively added. After stirring for 1 h, the solution was neutralized by the addition of triethylamine and concentrated to a syrupy residue that was purified by chromatography (65:35, *n*-hexane–EtOAc) to afford **S7** (0.245 g, 78% over three steps) as a thick syrup; R_f 0.23 (65:35, *n*-hexane–EtOAc); $[\alpha]_D$ +170.48 (*c* 0.39, $CHCl_3$); ^{1}H NMR (500 MHz, $CDCl_3$, δ_H) 7.46–7.30 (m, 10H), 5.40 (s, 1H, PhC*H*), 5.15 (d, *J* = 3.5 Hz, 1H, H-1), 4.98 (d, *J* = 3.6 Hz, 1H, H-1), 4.80 (d, *J* = 11.6 Hz, 1H), 4.70 (d, *J* = 11.6 Hz, 1H), 4.36 (d, *J* = 2.8 Hz, 1H), 4.18 (dd, *J* = 10.6, 3.4 Hz, 1H), 4.14 (s, 2H), 3.96 (dd, *J* = 10.9, 2.8 Hz, 1H), 3.89 (s, 3H), 3.73–3.60 (m, 7H), 3.49 (dt, *J* = 9.7, 6.4 Hz, 1H), 3.35 (dd, *J* = 13.0, 1.7 Hz, 1H), 2.32 (t, *J* = 7.5 Hz, 2H), 1.68–1.56 (m, *J* = 6.8 Hz, 4H), 1.41–1.31 (m, 8H); ^{13}C NMR (125 MHz, $CDCl_3$, δ_C) 174.48 (C=O), 137.42, 137.33, 129.31, 128.63, 128.30, 127.97, 127.01, 126.21, 101.01 (Ph*C*H), 99.48 (C-1), 98.04 (C-1), 77.32, 77.11, 76.90, 75.95, 75.48, 72.90, 71.80, 70.82, 68.83, 68.47, 67.42, 62.83, 61.11, 60.39, 59.75, 51.52, 34.06, 29.35, 29.11, 29.06, 29.00, 25.99, 24.87; HRMS (ESI) calcd. for $(M+Na)^+$ $C_{36}H_{48}NaN_6O_{11}$ 763.3273, found 763.3271.

8-Methoxycarbonyloctyl 3-*O*-benzyl-2-azido-2-deoxy-α-D-galactopyranosyl-(1→4)-3,6-di-*O*-benzyl-2-azido-2-deoxy-α-D-galactopyranoside (S8). To a solution of **S7** (0.24 g, 0.32 mmol) in dry DMF (3 mL) cooled to 0 °C under argon, sodium hydride (60% dispersion in mineral oil, 60 mg, 1.5 mmol) was added in portions followed by benzyl bromide (0.2 mL, 1.7 mmol) dropwise. The reaction mixture was allowed to warm to room temperature and stirred for 2 h before CH_3OH (0.1 mL) was added dropwise at 0 °C. The mixture was diluted with CH_2Cl_2 (25 mL) and washed with brine (15 mL) and water (15 mL). The CH_2Cl_2 layer was separated, dried with anhydrous Na_2SO_4 and concentrated to a syrupy residue that was re-dissolved in CH_2Cl_2–CH_3OH (1:1, 13 mL) followed by the addition of *p*-toluenesulfonic acid (TSA, 90 mg, 0.47 mmol) and water (0.2 mL). The reaction mixture was then stirred for 48 h, neutralized by the dropwise addition of triethylamine and concentrated to a syrupy residue that was purified by chromatography (3:2, *n*-hexane–EtOAc) to afford **S8** (0.218 g, 81% over two steps) as a thick syrup; R_f 0.17 (65:35, *n*-hexane–EtOAc); $[\alpha]_D$ +152.27 (*c* 0.2, $CHCl_3$); ^{1}H NMR (500 MHz, $CDCl_3$, δ_H) 7.47–7.30 (m, 15H), 5.03 (d, *J* = 3.6 Hz, 1H, H-1), 4.98 (d, *J* = 3.6 Hz, 1H, H-1), 4.85 (d, *J* = 11.7 Hz, 1H), 4.76–4.65 (m, 3H), 4.63–4.53 (m, 2H), 4.33 (d, *J* = 2.8 Hz, 1H), 4.18–4.09 (m, 2H), 4.03–3.93 (m, 3H), 3.92 (t, *J* = 2.8 Hz, 1H), 3.73–3.64 (m, 6H), 3.59 (dd, *J* = 8.4, 5.1 Hz, 1H), 3.53–3.45 (m, 2H), 3.45–3.39 (m, 1H), 2.83 (br. s, 1H), 2.32 (t, *J* = 7.5 Hz, 2H), 2.00 (br. s, 1H), 1.69–1.58 (m, 4H), 1.40–

1.27 (m, 8H); ^{13}C NMR (125 MHz, $CDCl_3$, δ_C) 174.30 (C=O), 137.48, 137.42, 137.11, 128.67, 128.62, 128.55, 128.26, 128.16, 128.10, 128.05, 128.02, 127.30, 98.65 (C-1), 98.07 (C-1), 77.37, 77.12, 76.86, 75.99, 75.75, 73.63, 72.54, 72.09, 71.76, 69.16, 69.00, 68.54, 67.52, 67.09, 62.68, 59.74, 59.35, 51.46, 51.41, 34.09, 29.37, 29.16, 29.07, 26.03, 24.93; HRMS (ESI) calcd. for $(M+Na)^+$ $C_{43}H_{56}NaN_6O_{11}$ 855.3899, found 855.3896.

8-Methoxycarbonyloctyl 6-*O*-benzoyl-3-*O*-benzyl-2-azido-2-deoxy-α-D-galactopyranosyl-(1→4)-3,6-di-*O*-benzyl-2-azido-2-deoxy-α-D-galactopyranoside (S9). To a solution of **S8** (0.215 g, 0.26 mmol) in CH_2Cl_2 (8 mL) was added triethylamine (0.5 mL, 3.6 mmol), followed by benzoic anhydride (0.16 g, 0.7 mmol) and the mixture was stirred for 48 h. The solution was then concentrated and the residue was purified by column chromatography (3:1, *n*-hexane–EtOAc) to afford **S9** (0.209 g, 87%) as a thick syrup; R_f 0.3 (3:1, *n*-hexane–EtOAc); $[\alpha]_D$ +130.97 (*c* 0.2, $CHCl_3$); ^{1}H NMR (500 MHz, $CDCl_3$, δ_H) 7.93–7.87 (m, 2H), 7.62–7.06 (m, 18H), 5.05 (d, *J* = 3.6 Hz, 1H, H-1), 5.00 (d, *J* = 3.6 Hz, 1H, H-1), 4.88 (d, *J* = 11.7 Hz, 1H), 4.76 (d, *J* = 11.2 Hz, 1H), 4.73–4.65 (m, 2H), 4.63 (d, *J* = 11.7 Hz, 1H), 4.57 (d, *J* = 11.8 Hz, 1H), 4.50 (d, *J* = 5.9 Hz, 2H), 4.37 (d, *J* = 2.8 Hz, 1H), 4.08 (d, *J* = 3.1 Hz, 1H), 4.04–3.90 (m, 5H), 3.76–3.65 (m, 6H, OCH_3, included in multiplet), 3.61 (dd, *J* = 8.0, 4.7 Hz, 1H), 3.49 (dt, *J* = 9.7, 6.6 Hz, 1H), 2.52 (br. S, 1H), 2.33 (t, *J* = 7.5 Hz, 2H), 1.70–1.58 (m, 4H), 1.40–1.28 (m, 8H); ^{13}C NMR (125 MHz, $CDCl_3$, δ_C) 174.31 (C=O), 165.95 (C=O), 137.40, 137.26, 137.17, 133.44, 133.06, 130.13, 129.84, 129.76, 128.67, 128.59, 128.49, 128.46, 128.28, 128.25, 128.18, 128.15, 128.06, 127.81, 127.22, 98.53 (C-1), 98.16 (C-1), 77.37, 77.11, 76.86, 76.03, 75.39, 73.64, 72.35, 72.09, 72.05, 69.10, 68.55, 67.63, 67.07, 65.39, 62.12, 59.68, 59.33, 51.48, 34.11, 29.74, 29.38, 29.18, 29.09, 26.04, 24.95; HRMS (ESI) calcd. for $(M+Na)^+$ $C_{50}H_{60}N_6O_{12}Na$ 959.4161, found 959.4168.

8-Methoxycarbonyloctyl 3,4,6-tri-*O*-acetyl-2-azido-2-deoxy-α-D-galactopyranosyl-(1→4)-6-*O*-benzoyl-3-*O*-benzyl-2-azido-2-deoxy-α-D-galactopyranosyl-(1→4)-3,6-di-*O*-benzyl-2-azido-2-deoxy-α-D-galactopyranoside (S10). A mixture of trichloroacetimidate **S1**[1] (prepared from 0.61 g, 1.84 mmol of the hemiacetal), acceptor **S9** (0.545 g, 0.58 mmol) and molecular sieves (4Å, 0.6 g) in dry CH_2Cl_2–Et_2O (1:1, 30 mL) was stirred under argon for 30 min., cooled to −15 °C and then TBSOTf (35 μL) was added dropwise. The mixture was stirred initially at −10 °C for 20 min and then warmed to 0 °C over 1 h before the acid was quenched by the addition of a few drops of triethylamine. The mixture was filtered through a pad of Celite and washed with CH_2Cl_2 (30 mL). The combined filtrate was concentrated and the residue was purified by chromatography (3:1, *n*-hexane–EtOAc) to afford **S10** (0.385 g, 53%) as a white foam; R_f 0.23 (3:1, *n*-hexane–EtOAc); $[\alpha]_D$ +193.04 (*c* 0.13, $CHCl_3$); ^{1}H NMR (500 MHz, $CDCl_3$, δ_H) 7.90–7.85 (m, 2H), 7.66–7.59 (m, 1H), 7.51–7.26 (m, 15H), 7.20–7.12 (m, 1H), 7.00–6.93 (m, 1H), 5.42 (dd, *J* = 3.2, 1.5 Hz, 1H), 5.35 (dd, *J* = 11.1, 3.2 Hz, 1H), 5.06 (d, *J* = 3.7 Hz, 1H, H-1), 4.96 (d, *J* = 3.5 Hz, 1H, H-1), 4.92 (d, *J* = 3.5 Hz, 1H, H-1), 4.85 (dd, *J* = 11.9, 10.0 Hz, 2H), 4.75 (d, *J* = 11.7 Hz, 1H), 4.69 (d, *J* = 12.0 Hz, 1H), 4.63 (d, *J* = 11.8 Hz, 1H), 4.60–4.53 (m, 3H), 4.52 (d, *J* = 2.1 Hz, 1H), 4.46 (dd, *J* = 10.2, 4.9 Hz, 1H), 4.37 (d, *J* = 2.9 Hz, 1H), 4.15 (d, *J* = 2.7 Hz, 1H), 4.06–3.94 (m, 3H), 3.93–3.86 (m, 3H), 3.75 (dd, *J* = 11.1, 3.5 Hz, 1H), 3.70–3.55 (m, 7H, OCH_3, included in multiplet), 3.50–3.42 (m, 2H), 2.32 (t, *J* = 7.5 Hz, 2H), 2.25–2.15 (m, 1H), 2.07 (s, 3H, $COCH_3$), 2.06 (s, 3H, $COCH_3$), 1.88 (s, 3H, $COCH_3$), 1.69–1.56 (m, 4H), 1.37–1.29 (m, 8H); ^{13}C NMR (125 MHz, $CDCl_3$, δ_C) 174.29 (C=O), 170.02 (C=O), 170.00 (C=O), 169.86 (C=O), 165.14 (C=O), 137.23, 137.10, 137.00, 133.21, 129.66, 129.63, 128.64, 128.56, 128.47, 128.45, 128.38, 128.21, 128.13, 127.98, 127.74, 127.53, 127.03, 99.32 (C-1), 98.30 (C-1), 98.07 (C-1), 77.33, 77.07, 76.82, 75.28, 75.23, 74.38, 73.70, 72.51, 72.12, 72.04, 69.02, 68.74, 68.52, 68.31, 68.24, 67.22, 67.14, 66.82, 66.68, 61.16, 60.36, 59.86, 59.82, 58.31, 51.46, 34.10, 29.72, 29.36, 29.16, 29.08, 26.02, 24.94, 20.70, 20.58, 20.54, 20.41; HRMS (ESI) calcd. for $(M+Na)^+$ $C_{62}H_{75}NaN_9O_{19}$ 1272.5071, found 1272.5108.

8-Methoxycarbonyloctyl 3,4,6-tri-*O*-benzyl-2-azido-2-deoxy-α-D-galactopyranosyl-(1→4)-3,6-di-*O*-benzyl-2-azido-2-deoxy-α-D-galactopyranosyl-(1→4)-3,6-di-*O*-benzyl-2-azido-2-deoxy-α-D-galactopyranoside (S11). To a solution of compound **S10** (0.29 g, 0.23 mmol) in CH_2Cl_2–CH_3OH (7 mL:2 mL) was added sodium methoxide in CH_3OH to bring the pH of the reaction mixture to 8–9. The reaction mixture was stirred overnight and then neutralized by the addition of pre-washed Amberlite IR 120 H^+ resin, filtered and concentrated to a syrupy residue that was purified by column chromatography (2:3, *n*-hexane–EtOAc, followed by 9:1, CH_2Cl_2–CH_3OH) to obtain the corresponding tetra-ol (209 mg, 88%) as a thick syrup; R_f 0.55, (9:1, CH_2Cl_2–CH_3OH). The compound was then dried overnight under vacuum, redissolved in dry DMF (3 mL) and the solution cooled to 0 °C. Sodium hydride (60% dispersion in mineral oil, 80 mg, 2 mmol) was added in portions followed by benzyl bromide (0.2 mL, 1.7 mmol) dropwise. The reaction mixture was allowed to warm to room temperature and stirred for 4 h before CH_3OH (0.2 mL) was added dropwise at 0 °C. The mixture was diluted with CH_2Cl_2 (25 mL) and washed with brine (15 mL) and water (15 mL). The CH_2Cl_2 layer was separated, dried with anhydrous Na_2SO_4 and concentrated to a syrupy residue that was purified by chromatography (85:15, *n*-hexane–EtOAc), to afford **S11** (0.25 g, 88% over two steps) as a thick syrup; R_f0.19, (85:15, *n*-hexane–EtOAc), ^1H NMR (500 MHz, $CDCl_3$, δ_H) 7.48–7.12 (m, 35H), 5.80 (d, J = 3.7 Hz, 1H, H-1), 5.10 (d, J = 3.7 Hz, 1H, H-1), 4.94 (d, J = 3.7 Hz, 1H, H-1), 4.97–4.88 (m, 2H), 4.83 (d, J = 11.0 Hz 1H), 4.78 (d, J = 11.0 Hz, 1H), 4.61 (d, J = 11.0 Hz, 1H), 4.61–4.48 (m, 5H), 4.38–4.30 (m, 4H), 4.10–3.80 (m, 10H), 3.74–3.43 (m, 8H, OCH_3 included in multiplet), 3.18 (dd, J = 5.3, 8.2 Hz, 1H), 3.08 (dd, J = 5.0, 8.2 Hz, 1H), 2.33 (t, J = 7.5 Hz, 2H), 1.68–1.57 (m, 4H), 1.37–1.30 (m, 8H); ^{13}C NMR (125 MHz, $CDCl_3$, δ_C) 174.30 (C=O), 138.62, 137.99, 137.79, 137.77, 137.67, 137.61, 137.53, 128.58, 128.47, 128.37, 128.31, 128.22, 128.20, 128.10, 128.06, 127.95, 127.84, 127.81, 127.75, 127.66, 127.58, 127.53, 127.47, 127.10, 127.01, 99.04, (C-1), 98.98 (C-1), 98.13 (C-1), 77.34, 77.27, 77.08, 76.83, 76.02, 75.88, 74.93, 73.65, 73.09, 73.07, 72.94, 72.61, 72.08, 71.85, 71.79, 71.62, 69.26, 69.21, 69.16, 68.49, 67.57, 67.16, 66.46, 60.23, 60.06, 59.65, 51.48, 34.12, 29.39, 29.18, 29.10, 26.04, 24.96; HRMS (ESI) calcd. for $(M+Na)^+$ $C_{77}H_{89}NaN_9O_{15}$ 1402.6370, found 1402.6377.

8-Methoxycarbonyloctyl (2-N-acetylamino-2-deoxy-α-D-galactopyranosyl)-(1→4)-(2-N-acetylamino-2-deoxy-α-D-galactopyranosyl)-(1→4)-2-N-acetylamino-2-deoxy-α-D-galactopyranoside (S12). To a solution of **S11** (0.25 g, 0.18 mmol) in THF (12 mL) was added pyridine (1 mL), water (1 mL) and triphenylphosphine (0.3 g, 1.14 mmol) and the mixture was stirred for 18 h. The solution was then concentrated, co-evaporated with toluene (2 × 10 mL) and dried under high vacuum for 1 h. About half of the syrupy residue was then re-dissolved in CH_2Cl_2 (5 mL) and pyridine (3.5 mL) was added before cooling to 0 °C. Acetic anhydride (1.25 mL) was added dropwise, the cooling removed and the mixture stirred overnight. To the solution, CH_3OH (2 mL) was added dropwise at 0 °C and the mixture was stirred for 30 min at room temperature before being diluted with CH_2Cl_2 (40 mL). The CH_2Cl_2 solution was then washed with a10% aqueous copper sulfate solution (3 × 35 mL), water (25 mL), dried with anhydrous Na_2SO_4, filtered and concentrated to a syrupy residue that was purified on a silica gel column (1:4, *n*-hexane–EtOAc). The fractions containing the product were combined, concentrated, and the residue was then re-dissolved THF–EtOAc–CH_3OH (1:1:1, 15 mL), and then 10% Pd-C (70 mg) was added. After stirring under a hydrogen atmosphere for 18 h, the mixture was filtered to remove the catalyst, washed with THF–CH_3OH (1:1, 16 mL) and concentrated to a syrupy residue that was re-dissolved in water (10 mL) and then washed with CH_2Cl_2 (3 × 6 mL). The aqueous layer was separated, concentrated to half the volume, and lyophilized to obtain the title compound **S12** (58 mg, 80% over three steps) as a fluffy solid. ^1H NMR (500 MHz, CD_3OD, δ_H) 5.08–5.05 (m, 2H, 2 × H-1), 5.02 (d, J = 4.9 Hz, 1H, H-1), 4.50–4.41 (m, 2H), 4.40–4.35 (m, 1H), 4.32–4.15 (m, 4H), 4.25 (dd, J = 11.3, 3.6 Hz, 1H), 4.05 (ddd, J = 15.9, 3.1, 1.0 Hz, 2H), 3.99–3.91 (m, 2H), 3.87 (ddd, J = 11.2, 6.5, 2.9 Hz, 3H), 3.83–3.67 (m, 6H), 3.76 (s, 3H, OCH_3 merged), 2.45 (dd, J = 2.5, 2.5 Hz, 2H), 2.16 (s, 3H,

$COCH_3$), 2.14 (s, 3H, $COCH_3$), 2.11 (s, 3H, $COCH_3$), 1.67–1.54 (m, 4H), 1.39 (d, *J* = 7.5 Hz, 1H), 1.33 (d, *J* = 3.2 Hz, 7H); ^{13}C NMR (125 MHz, CD_3OD, δ_C) 178.83 (C=O), 175.54 (C=O), 175.52 (C=O), 175.46 (C=O), 100.78 (C-1), 100.49 (C-1), 98.92 (C-1), 79.67, 78.99, 72.86, 72.51, 70.36, 69.55, 69.29, 69.23, 69.08, 62.75, 61.42, 61.33, 52.34, 52.09, 52.02, 51.88, 49.54, 49.37, 49.20, 49.03, 48.86, 48.69, 48.52, 34.79, 30.51, 30.35, 30.33, 30.13, 27.22, 26.01, 22.77, 22.72, 22.70; HRMS (ESI) calcd. for $(M+Na)^+$ $C_{34}H_{59}N_3O_{18}Na$ 820.3686, found 820.3683.

8-Carboxyoctyl (2-N-acetylamino-2-deoxy-α-D-galactopyranosyl)-(1→4)-(2-N-acetylamino-2-deoxy–α-D-galactopyranosyl)-(1→4)-2-N-acetylamino-2-deoxy-α-D-galactopyranoside (S13). To a solution of **S12** (25 mg, 0.031 mmol) was added aqueous sodium hydroxide [2.8 mg (0.07 mmol) in 1.4 mL H_2O] and the mixture was stirred for 1 h. The reaction mixture was then neutralized by the addition of pre-washed Amberlite IR 120 H^+ resin, filtered and lyophilized to obtain the **S13** (25 mg, quantitative) as a fluffy solid, which was then directly used for the coupling reaction with propargylamine. HRMS (ESI) calcd. for $(M-H)^-$ $C_{33}H_{56}N_3O_{18}$ 782.3564, found 782.3560.

8-Propargylaminocarbonyloctyl (2-N-acetylamino-2-deoxy-α-D-galactopyranosyl)-(1→4)-(2-N-acetylamino-2-deoxy–α-D-galactopyranosyl)-(1→4)-2-N-acetylamino-2-deoxy-α-D-galactopyranoside (S14) To a solution of **S13** (23 mg, 0.029 mmol), propargylamine (8 μL, 0.15 mmol) and DIPEA (18 μL, 0.1 mmol) in DMF (1.4 mL) was added TBTU (33 mg, 0.1 mmol) and the solution was stirred for 36 hours. The reaction mixture was then concentrated (<50 °C) to a syrup that was re-dissolved in water and purified by C-18 chromatography to obtain the title compound **S14** (24 mg, quantitative) as a fluffy solid after lyophilization. ^{1}H NMR (500 MHz, D_2O, δ_H) 5.08–5.06 (m, 2H, 2 × H-1), 5.02 (d, *J* = 3.7 Hz, 1H, H-1), 4.48 (dd, *J* = 6.6 Hz, 1H), 4.43 (dd, *J* = 9.1 Hz, 1H), 4.37 (dd, *J* = 11.4, 3.8 Hz, 1H), 4.29 (ddd, *J* = 11.3, 3.8, 1.2 Hz, 2H), 4.22 (dd, *J* = 11.4, 2.8 Hz, 1H), 4.18 (d, *J* = 2.8 Hz, 1H), 4.15–4.05 (m, 5H), 4.02 (d, *J* = 2.4 Hz, 2H), 3.84–3.75 (m, 5H), 3.75–3.67 (m, 2H), 3.57 (dt, *J* = 10.2, 6.2 Hz, 1H), 2.88 (s, 1H), 2.67 (t, *J* = 2.5 Hz, 1H, -CC*H*), 2.32 (t, *J* = 7.3 Hz, 2H), 2.16 (s, 3H, $COCH_3$), 2.15 (s, 3H, $COCH_3$), 2.11 (s, 3H, $COCH_3$), 1.71–1.60 (m, 4H), 1.45–1.34 (m, 8H); ^{13}C NMR (125 MHz, D_2O, δ_C) 178.16 (C=O), 175.59 (C=O), 175.55 (C=O), 175.49 (C=O), 126.36, 99.29 (C-1), 99.20 (C-1), 97.79 (C-1), 80.76, 77.87, 77.41, 72.62, 72.52, 72.39, 71.71, 69.28, 69.21, 68.20, 68.12, 67.59, 61.49, 61.47, 60.66, 51.34, 51.26, 51.12, 39.00, 36.53, 29.64, 29.61, 29.35, 29.28, 29.19, 29.01, 26.14, 22.93, 22.91, 22.88; HRMS (ESI) calcd. for $(M+Na)^+$ $C_{36}H_{60}N_4O_{17}Na$ 843.3846, found 843.3845.

BSA Conjugate of S14. A BSA conjugate was prepared by first conversion of the amino groups in BSA to azides (giving azido-BSA) and then using an azide–alkyl cycloaddition with trisaccharide **S14** and subsequent purification as described.[3] The loading of the trisaccharide on the BSA was 17/protein.

Table S1: Primers used in this study

Primers	Sequence*
**Allelic exchange vectors**	
KanR F	GGTCCGATAAACCCAGCGA
KanR R	CGATACAAATTCCTCGTAGGC
PelD up F	GGG**TCTAGA**TGGCTATTTTTTGGAGACGGG
PelD up R	TCGCTGGGTTTATCGGACCCCCTGTATTTCCAAATAGTAGAG
PelD down F	GCCTACGAGGAATTTGTATCGACTCCTAATGGCATCAAATATGG
PelD downR	GGG**GGATCC**ACAAAACTGCAGCTAATAAAAGAAA
PelE up F	GTAG**GGATCC**CATCTGGAATTCAATCGTTTAAAAGC
PelE up R	GTTCGCTGGGTTTATCGGACCTGCCAAATCTAAAATCACACAGAATAG
PelE down F	CCTACGAGGAATTTGTATCGTCAAAAGAGAACCGAGAATATTTAGCT
PelE downR	GTCA**CTCGAG**CAGATAACTTCAATAAATCTGGCC
PelA up F	GTAG**GGATCC**ATCATCAAGCATTTGTCAACCTC
PelA up R	GTTCGCTGGGTTTATCGGACCCTCCCTTGAATAAGCCAATACC
PelA down F	CCTACGAGGAATTTGTATCGGCTACTTCTGCAAAAATAGAAATTG
PelA downR	GTCA**CTCGAG**GAACATTGGGCATCTGGGAG
PelF up F	GGGGAGCTCTATTTAGACGATTTTCCTTCCCCT
PelF up R	GTTCGCTGGGTTTATCGGACCACCCTCTAAAACCAAACAGATTCT
PelF down F	CCTACGAGGAATTTGTATCGAGACAATTATATAAGGAGTATGTAAGA
PelF down R	AAGGGGCATGCGTAAAACTGTCAACAGAACAATTC
PelG up F	GTAG**GGATCC**CATACTATCCGATCTATGCTTTTTCC
PelG up R	GTTCGCTGGGTTTATCGGACCTCGCAGTTCGAATCCTATCC
PelG downF	CCTACGAGGAATTTGTATCGGTGACTATTTTGAAAGCGAGGTC
PelG downR	GTCA**CTCGAG**CCATCAATATTTGAAAGGCCAACC
1592 up F	GTAG**GGATCC**GGTCTAACAAAGACAAGTGTTACG
1592 up R	GTTCGCTGGGTTTATCGGACCTGTAAAATTTTCCGATCTCAAAACCG
1592 downF	CCTACGAGGAATTTGTATCGCAGTATAATGGAGAGTATCACGG
1592 downR	GTCA**CTCGAG**GGACTTCAGCAATTTTAGGC
1593 up F	GTAG**CATATG**TTCGAAAGGAAAATGATCCTAGG
1593 up R	GTTCGCTGGGTTTATCGGACCCTGTTCTTTTTTATTTGCTTTCATAGAC
1593 downF	CCTACGAGGAATTTGTATCGCATGATTTTGAATATTGGTATGATACAGATC
1593 downR	GTCA**CTCGAG**CGTTTTCCAATCATAGACTCC
1594 up F	CTA**GTCGAC**GAAAGATTATCGTAGTATTCTAAAAACCTTTGCCAT
1594 up R	GTTCGCTGGGTTTATCGGACCTAAAAATTTGATCCACTTATTATCCATCTTTTGACC
1594 downF	GCGCCTACGAGGAATTTGTATCGATAAAGGTTGATGTCTATGAAAGCAAATAAAAAAGAAC
1594 downR	CTA**GGTACCG**ATTCGAACTAATGGCCAAAGTAACCATAGC
**Sequencing Primers**	
PelD F	CCA AGC TTG CAT GCC TGC AG
PelD R	CAG TCA CGA CGT TGT AAA ACG AC
PelE F	GGCACCGTTATGGCTGGTTT
PelE R	GCAGTTACATGACCTGATGTATCATC
PelA F	ATGATTGGTGGAGCAACAGC
PelA R	TGCTAAAGCATAACATTCTTCTGC
PelF F	TATGAAAGAGCAGTTCGCGGTAT
PelF R	ACCCCATAGCCTAAACAAAGTG
PelG F	TTTTCGACCTCCTTCAAGAAGG
PelG R	TATACCAACGTTTCCCATGTAGG
1594 F	CTTGATCTTGTTTAGTGAACTATG
1594 R	ATCCTTAACTGCTGTCCGGT
1593 F	GCTCTTGAAGCACATGGACTAG
1593 R	CCTTCTTTGCGCCAGCGATT
1592 F	TCATGTGGAGTTAGCTAAAGATCC
1592 R	GTAACTGACAGTGTAAGCTTAGTGG
1591 F	AGTCCTCATGCCTTGTGTCGT
1591 R	GCAAAACCAGCCAAAAGAGCACTC
T7	TAATACGACTCACTATAGGG
T7 ter	GCTAGTTATTGCTCAGCGG

Plasmid Vectors	
pET28a	IPTG inducible protein expression vector- Kanamycin resistant
pCC1-4k	Plasmid vector used by Biobasic for inserting SIR_1591 mutant allele- Chloramphenicol resistant
pET28a-SIR1591$^{28-468}$	SIR_1591 encoding residues 28-468 in pET28a vector. Expressed thrombin-cleavable fusion protein MGSSH6SSGLVPRGSHM-SIR_1591 residues 28-468 in pET-28a.

* Restriction sites are indicated in bold and overlap with Kan cassette in the allelic exchange vectors are underlined

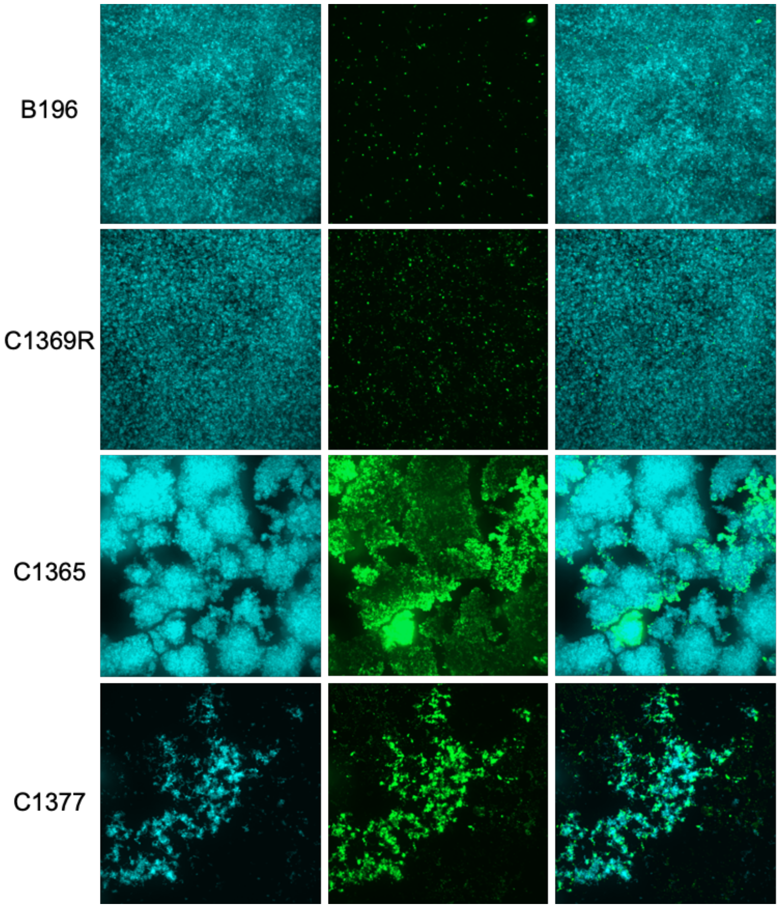

Supplementary Figure S1. **Pel does not significantly affect surface adherence in *S. intermedius.*** Immunostaining for presence of Pel in four of the eleven clinical strains. B196 and C1369R do not make Pel but are surface adherent while C1365 and C1377 are positive for Pel staining in aggregates. Pel was detected using a primary mouse anti-(GalNAc)$_3$ antibody followed by secondary anti-mouse Alexafluor488 (Green) and DAPI to stain for bacterial cells (Blue). Confocal images were taken using a Spinning disk confocal microscope at 25x magnification.

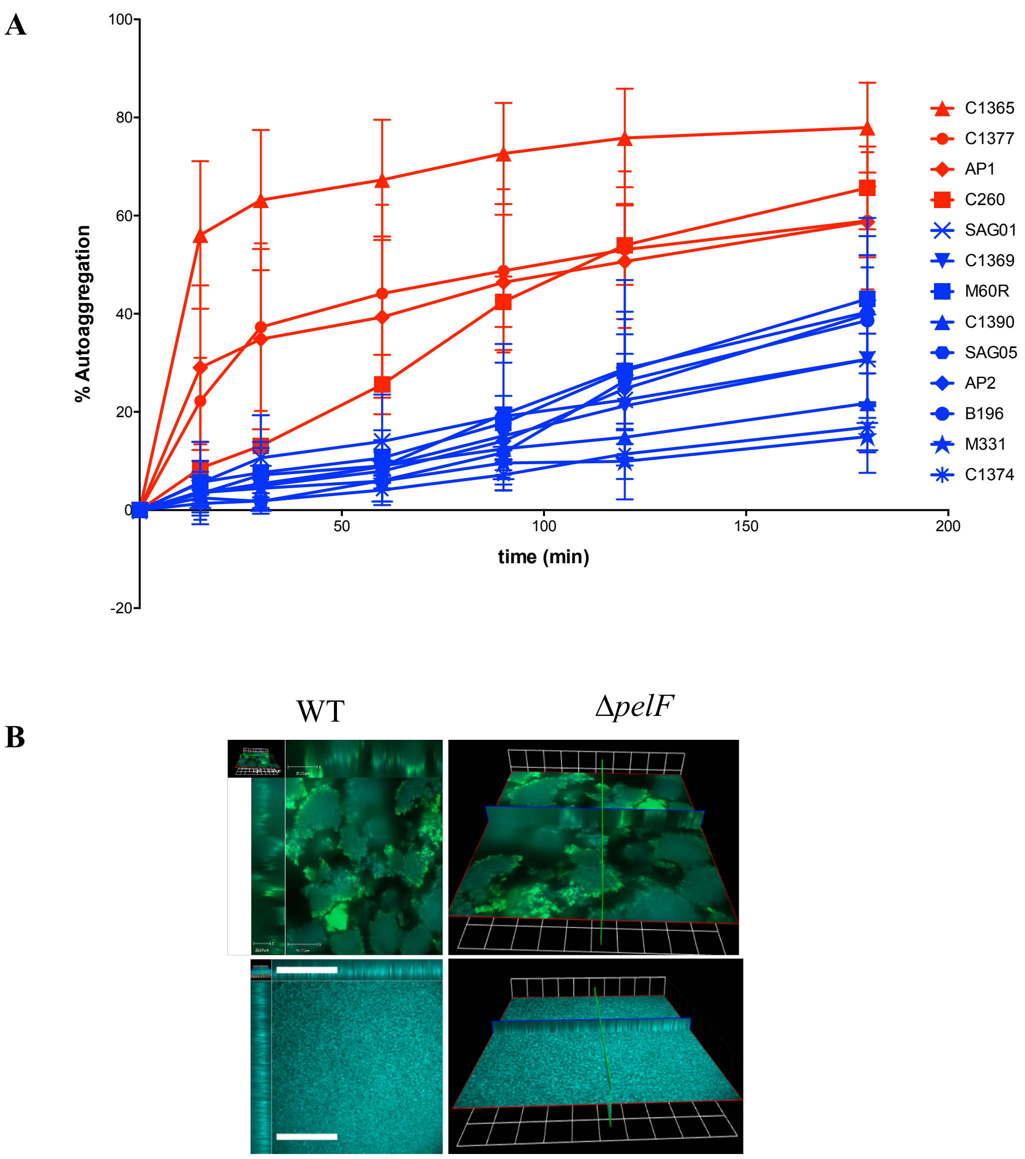

Supplementary Figure S2. Pel biosynthesis contributes to auto-aggregation in hyper-aggregating strains of *S. intermedius*. A. *S. intermedius* strains were screened over time for auto-aggregation capacity. Four hyper-aggregating strains were identified (red). **B.** Representative XYZ planes and 3D planes from confocal images of C1365 WT and Δ*pelF*.

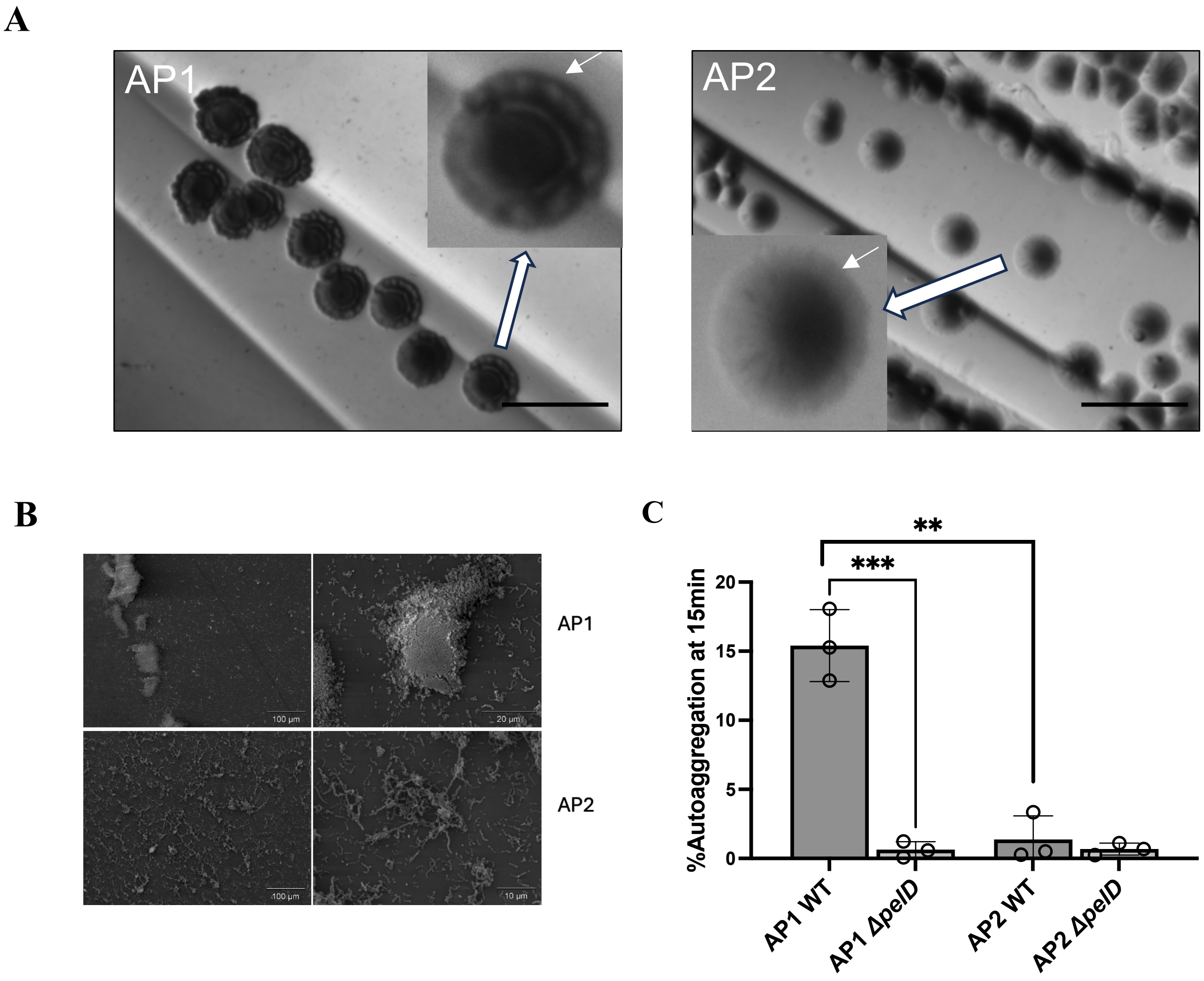

Supplementary Figure S3: Truncation of *pelD* gene in AP2 results in a loss of the aggregation phenotype. (**A**) Colony morphology of clinical isolates, AP1 and AP2, observed under a stereoscopic microscope. The strains were streaked onto THY-congo red agar and incubated at 37 ºC with 5% CO_2 for 3 days. AP1 displays a wrinkly phenotype while AP2 has a smoother surface. The scale bar is equivalent to 5 mm. Inset shows a zoomed in colony in each panel with the small white arrows indicating the wrinkly vs smooth edges in AP1 and AP2 respectively. (**B**) Scanning electron micrographs of AP1 and AP2. AP1 forms tight aggregates while Pel- lacking AP2 bacteria are more spread out and do not aggregate.
(C) Aggregation assay comparing phenotypes of the clinical isolates AP1 and AP2 at 15 min. AP1 wild- type (WT) auto-aggregates to a greater extent than WT AP2. Deletion of *pelD* (Δ*pelD*) in AP1 results in a decrease of auto-aggregation compared to the AP1 WT strain at 15 min. Low auto-aggregation is observed in both the AP2 WT and AP2 Δ*pelD* strains. Error bars represent standard deviations from three independent samples. Statistical analyses were performed on 3 biological replicates using unpaired T test to compare each wild type strain to its derived mutant. AP1 and AP2 were compared separately using a T test. ** $=p \leq 0.01$, ***$= p<0.005$.

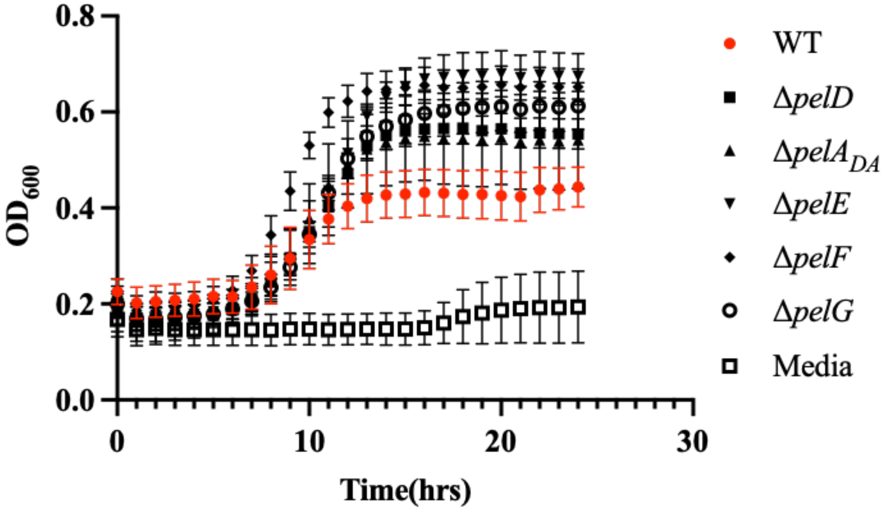

Supplementary Figure S4. Growth curves for wildtype and *pel* gene cluster mutant strains of *S. intermedius* C1365. *S. intermedius* wildtype strain C1365 (WT) and the corresponding mutants of the genes in the putative *pel* operon were grown in THY broth overnight at 37 ºC and 5% CO_2 in a 96 well plate. The plate was incubated in a SpectraMax i3X Multi-Mode Assay Microplate Reader. Optical density (600nm) was measured every hour over 24 h. There were no significant differences observed in the growth between the WT and the mutants.

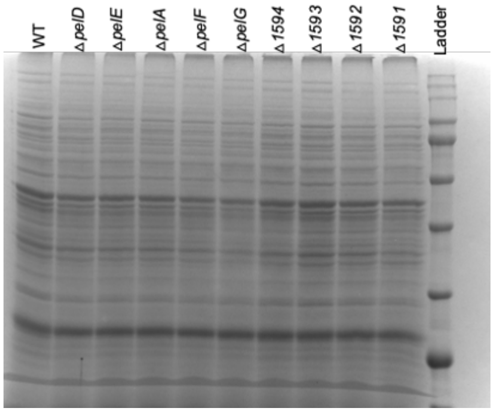

Supplementary Figure S5. SDS-PAGE analysis of WT and mutant lysates The coomassie stained SDS_PAGE gel shows equal protein loading across the samples normalized to OD 2 from Wild type C1365 strain and the corresponding Pel operon mutants.

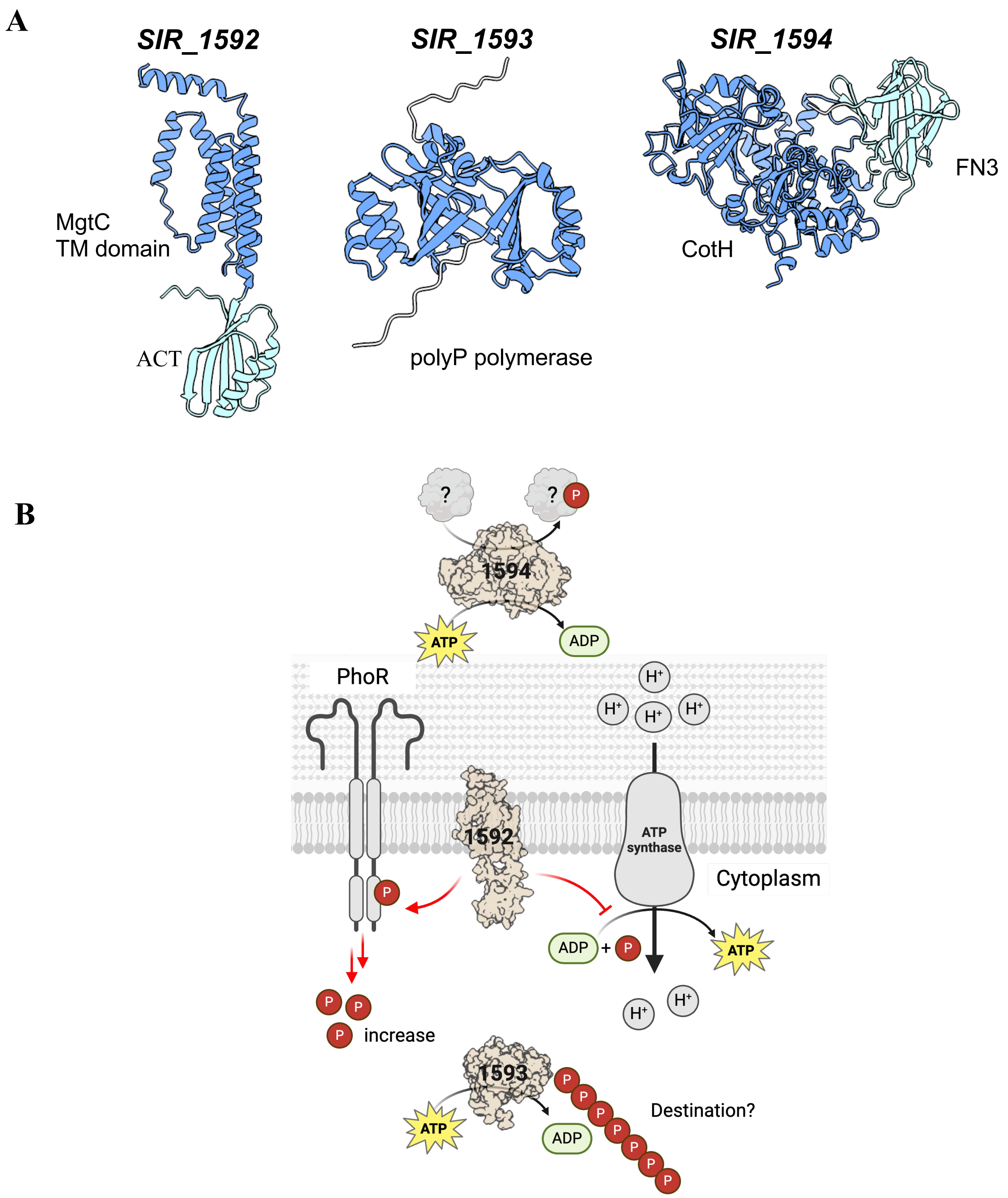

Supplementary Figure S6. AlphaFold models SIR_1592-1594. (A) Cartoon representation of AlphaFold models drawn to scale**.** Dark and light blue represents the N- and C-terminal domains of each protein, respectively. **(B)** Schematic depicting the surface representation of the AF2 model of SIR_1592-4 and potential roles of these genes in phosphate metabolism.

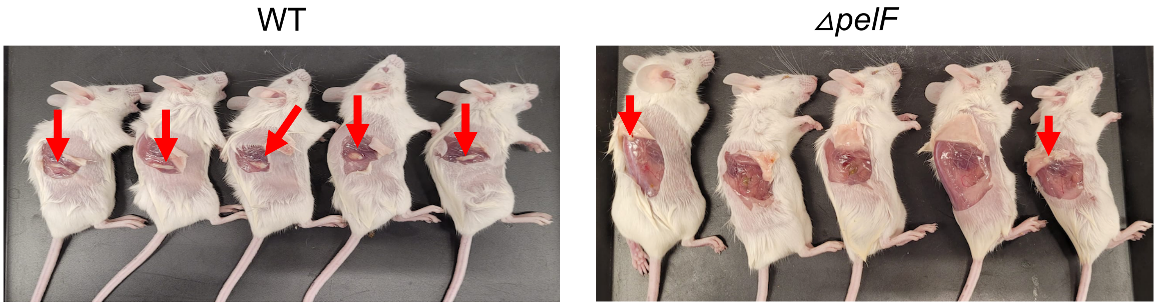

Supplementary Figure S7. Murine subcutaneous abscess model for pathogenicity of *S. intermedius* aggregates. Female Balb/C mice were infected with wildtype C1365 (WT) or with the corresponding PelF mutant (*∆pelF)*. Mice were sacrificed 6 days post infection and the abscesses measured for size, weight and bacterial burden. Red arrows show presence of abscess in the infected mice after 6 days.
